# Multiple Independent Loci at Chromosome 15q25.1 Affect Smoking Quantity: a Meta-Analysis and Comparison with Lung Cancer and COPD

**DOI:** 10.1371/journal.pgen.1001053

**Published:** 2010-08-05

**Authors:** Nancy L. Saccone, Robert C. Culverhouse, Tae-Hwi Schwantes-An, Dale S. Cannon, Xiangning Chen, Sven Cichon, Ina Giegling, Shizhong Han, Younghun Han, Kaisu Keskitalo-Vuokko, Xiangyang Kong, Maria Teresa Landi, Jennie Z. Ma, Susan E. Short, Sarah H. Stephens, Victoria L. Stevens, Lingwei Sun, Yufei Wang, Angela S. Wenzlaff, Steven H. Aggen, Naomi Breslau, Peter Broderick, Nilanjan Chatterjee, Jingchun Chen, Andrew C. Heath, Markku Heliövaara, Nicole R. Hoft, David J. Hunter, Majken K. Jensen, Nicholas G. Martin, Grant W. Montgomery, Tianhua Niu, Thomas J. Payne, Leena Peltonen, Michele L. Pergadia, John P. Rice, Richard Sherva, Margaret R. Spitz, Juzhong Sun, Jen C. Wang, Robert B. Weiss, William Wheeler, Stephanie H. Witt, Bao-Zhu Yang, Neil E. Caporaso, Marissa A. Ehringer, Tim Eisen, Susan M. Gapstur, Joel Gelernter, Richard Houlston, Jaakko Kaprio, Kenneth S. Kendler, Peter Kraft, Mark F. Leppert, Ming D. Li, Pamela A. F. Madden, Markus M. Nöthen, Sreekumar Pillai, Marcella Rietschel, Dan Rujescu, Ann Schwartz, Christopher I. Amos, Laura J. Bierut

**Affiliations:** 1 Department of Genetics, Washington University School of Medicine, St. Louis, Missouri, United States of America; 2 Department of Internal Medicine, Washington University School of Medicine, St. Louis, Missouri, United States of America; 3 Division of Biostatistics, Washington University School of Medicine, St. Louis, Missouri, United States of America; 4 Department of Psychiatry, University of Utah School of Medicine, Salt Lake City, Utah, United States of America; 5 Department of Psychiatry, Virginia Commonwealth University, Richmond, Virginia, United States of America; 6 Department of Human Molecular Genetics, Virginia Commonwealth University, Richmond, Virginia, United States of America; 7 Department of Genomics, Life and Brain Center, University of Bonn, Bonn, Germany; 8 Institute of Neuroscience and Medicine (INM-1), Research Center Jülich, Jülich, Germany; 9 Department of Psychiatry, University of Munich (LMU), Munich, Germany; 10 Departments of Psychiatry, Genetics, and Neurobiology, Yale University School of Medicine, New Haven, Connecticut, United States of America; 11 Department of Epidemiology, University of Texas M. D. Anderson Cancer Center, Houston, Texas, United States of America; 12 Department of Public Health, University of Helsinki, Helsinki, Finland; 13 Department of Quantitative Sciences, GlaxoSmithKline, King of Prussia, Pennsylvania, United States of America; 14 Division of Cancer Epidemiology and Genetics, National Institutes of Health, Bethesda, Maryland, United States of America; 15 Department of Public Health Sciences, University of Virginia, Charlottesville, Virginia, United States of America; 16 Department of Sociology, Brown University, Providence, Rhode Island, United States of America; 17 Environmental and Occupational Medicine and Epidemiology Program, Harvard School of Public Health, Boston, Massachusetts, United States of America; 18 Institute for Behavioral Genetics, University of Colorado, Boulder, Colorado, United States of America; 19 Epidemiology Research, American Cancer Society, Atlanta, Georgia, United States of America; 20 Department of Psychiatry, Washington University School of Medicine, St. Louis, Missouri, United States of America; 21 Section of Cancer Genetics, Institute of Cancer Research, Sutton Surrey, United Kingdom; 22 Karmanos Cancer Institute, Wayne State University, Detroit, Michigan, United States of America; 23 Department of Epidemiology, Michigan State University, East Lansing, Michigan, United States of America; 24 National Institute for Health and Welfare, Helsinki, Finland; 25 Department of Epidemiology, Harvard School of Public Health, Boston, Massachusetts, United States of America; 26 Department of Nutrition, Harvard School of Public Health, Boston, Massachusetts, United States of America; 27 Queensland Institute of Medical Research, Brisbane, Queensland, Australia; 28 Department of Psychiatry and Neurobehavioral Sciences, University of Virginia, Charlottesville, Virginia, United States of America; 29 Department of Otolaryngology and Communicative Sciences, The University of Mississippi Medical Center, Jackson, Mississippi, United States of America; 30 Institute for Molecular Medicine Finland FIMM, Helsinki, Finland; 31 Department of Medical Genetics, University of Helsinki, Helsinki, Finland; 32 Wellcome Trust Sanger Institute, Cambridge, United Kingdom; 33 Broad Institute of Harvard and Massachusetts Institute of Technology, Cambridge, Massachusetts, United States of America; 34 Department of Medicine (Genetics Program), Boston University School of Medicine, Boston, Massachusetts, United States of America; 35 Department of Human Genetics, Eccles Institute of Human Genetics, University of Utah School of Medicine, Salt Lake City, Utah, United States of America; 36 Information Management Services, Rockville, Maryland, United States of America; 37 Department of Genetic Epidemiology in Psychiatry, Central Institute of Mental Health Mannheim, Mannheim, Germany; 38 Department of Integrative Physiology, University of Colorado, Boulder, Colorado, United States of America; 39 Cambridge Biomedical Research Centre, Cambridge, United Kingdom; 40 Department of Biostatistics, Harvard University, Boston, Massachusetts, United States of America; 41 Institute of Human Genetics, University of Bonn, Bonn, Germany; 42 Glaxo SmithKline, Research Triangle Park, North Carolina, United States of America; 43 Department of Psychiatry, University of Bonn, Bonn, Germany; Georgia Institute of Technology, United States of America

## Abstract

Recently, genetic association findings for nicotine dependence, smoking behavior, and smoking-related diseases converged to implicate the chromosome 15q25.1 region, which includes the *CHRNA5-CHRNA3-CHRNB4* cholinergic nicotinic receptor subunit genes. In particular, association with the nonsynonymous *CHRNA5* SNP rs16969968 and correlates has been replicated in several independent studies. Extensive genotyping of this region has suggested additional statistically distinct signals for nicotine dependence, tagged by rs578776 and rs588765. One goal of the Consortium for the Genetic Analysis of Smoking Phenotypes (CGASP) is to elucidate the associations among these markers and dichotomous smoking quantity (heavy versus light smoking), lung cancer, and chronic obstructive pulmonary disease (COPD). We performed a meta-analysis across 34 datasets of European-ancestry subjects, including 38,617 smokers who were assessed for cigarettes-per-day, 7,700 lung cancer cases and 5,914 lung-cancer-free controls (all smokers), and 2,614 COPD cases and 3,568 COPD-free controls (all smokers). We demonstrate statistically independent associations of rs16969968 and rs588765 with smoking (mutually adjusted p-values<10^−35^ and <10^−8^ respectively). Because the risk alleles at these loci are negatively correlated, their association with smoking is stronger in the joint model than when each SNP is analyzed alone. Rs578776 also demonstrates association with smoking after adjustment for rs16969968 (p<10^−6^). In models adjusting for cigarettes-per-day, we confirm the association between rs16969968 and lung cancer (p<10^−20^) and observe a nominally significant association with COPD (p = 0.01); the other loci are not significantly associated with either lung cancer or COPD after adjusting for rs16969968. This study provides strong evidence that multiple statistically distinct loci in this region affect smoking behavior. This study is also the first report of association between rs588765 (and correlates) and smoking that achieves genome-wide significance; these SNPs have previously been associated with mRNA levels of *CHRNA5* in brain and lung tissue.

## Author Summary

Nicotine binds to cholinergic nicotinic receptors, which are composed of a variety of subunits. Genetic studies for smoking behavior and smoking-related diseases have implicated a genomic region that encodes the alpha5, alpha3, and beta4 subunits. We examined genetic data across this region for over 38,000 smokers, a subset of which had been assessed for lung cancer or chronic obstructive pulmonary disease. We demonstrate strong evidence that there are at least two statistically independent loci in this region that affect risk for heavy smoking. One of these loci represents a change in the protein structure of the alpha5 subunit. This work is also the first to report strong evidence of association between smoking and a group of genetic variants that are of biological interest because of their links to expression of the alpha5 cholinergic nicotinic receptor subunit gene. These advances in understanding the genetic influences on smoking behavior are important because of the profound public health burdens caused by smoking and nicotine addiction.

## Introduction

Smoking is associated with many different diseases. Lung cancer is the illness most identified with smoking, and its prevalence over time mirrors per capita tobacco consumption [1]. There has been a reduction in smoking in the United States, and a concomitant decline in the incidence of lung cancer is beginning to emerge. Nonetheless more people die from lung cancer each year than from any other cancer [2]. Chronic obstructive pulmonary disease (COPD), another serious lung disease largely attributable to smoking, is also among the leading causes of death.

Recently, genetic findings for nicotine dependence and smoking related diseases converged to implicate the chromosome 15q25.1 region, which includes the *CHRNA5-CHRNA3-CHRNB4* cluster of cholinergic nicotinic receptor subunit genes. The nicotine dependence locus tagged by the single nucleotide polymorphism (SNP) rs16969968 and correlates has been replicated for smoking related traits including cigarettes-per-day and heavy smoking [3]–[11], and has been reported as the most significant association genome-wide in very recent meta-analyses [12]–[14]. This locus has also been associated with risk for lung cancer and COPD in several genome-wide association studies (GWAS) [6], [15]–[18]. This represents an exciting overlap of genetic findings for nicotine dependence and smoking related diseases. Though different SNPs may be reported by each study, the high correlation between the associated SNPs (r^2^>0.8 with rs16969968) implies that these statistical signals tag the same locus in European-ancestry populations. The SNP rs16969968 results in an amino acid change (D398N) in the alpha5 receptor subunit protein and has been shown to affect receptor function [19].

Extensive genotyping of the *CHRNA5-CHRNA3-CHRNB4* region has provided potential evidence for at least two additional distinct signals for nicotine dependence [4], [7], [8], [20]. A second locus, tagged by rs578776, is associated with nicotine dependence and smoking in several samples of European-ancestry, with the minor allele protective in the sense that it is elevated in controls; rs578776 has only low correlation with rs16969968 in European-ancestry populations (r^2^ = 0.24 in the HapMap CEU panel), though the linkage disequilibrium (LD) coefficient |D'| is 1. A third important locus in this region is a group of highly correlated SNPs, tagged by rs588765, which are associated with mRNA levels of *CHRNA5* in brain tissue [21], [22] and lung tissue [23]–[25] from European-ancestry subjects. When rs16969968 and rs588765 (or correlates) are studied together, three common haplotypes are observed, each with distinct effects on risk [7], [22]. There are hints that other, less common variants (minor allele frequency (MAF)≤5%) also contribute to nicotine dependence in this region, including a fourth locus represented by rs12914008 which has shown a relatively strong odds ratio of 0.73 in European-American subjects [4].

With the support of the National Institute on Drug Abuse (NIDA), we formed the Consortium for the Genetic Analysis of Smoking Phenotypes (CGASP), which includes smoking, lung cancer, and COPD researchers, to enable the pursuit of several research goals. For this first analysis project we focused on the chromosome 15q25.1 region containing *CHRNA5-CHRNA3-CHRNB4*. Specifically, we focused on the four distinct loci discussed above, which have low correlation with each other and have demonstrated evidence for involvement in nicotine dependence. Analyses were undertaken to investigate two questions: first, are there multiple statistically distinct genetic loci in this region that exert independent effects on smoking, and second, are similar patterns of genetic risk shared across smoking, lung cancer, and COPD.

## Methods

### Ethics statement

This study was conducted according to the principles expressed in the Declaration of Helsinki and obtained informed consent from participants and approval from the appropriate institutional review boards.

### Samples and study design

All subjects included in these meta-analyses were current or former smokers of European ancestry. Results from 34 datasets, which include a total of 38,617 unrelated subjects who were assessed for cigarettes-per-day, contributed to the meta-analyses. Eight of the datasets were drawn from family-based studies and contributed only a subset of unrelated individuals to these analyses. Table 1 gives sample sizes and demographics of each participating study sample. Text S7.describes additional details for each dataset, including ascertainment criteria and genotyping methods, and documents that four datasets are also members of other consortia. All datasets contributed to the analyses of smoking. A subset of these 34 datasets also had information on lung cancer cases and lung-cancer-free smoker controls (6 datasets, N = 13,614 smokers) and/or COPD cases and COPD-free smoker controls (4 datasets, N = 6,182 smokers). The data for these traits are described in Table 2 and Table 3 respectively.

**Table 1:** Description of contributing datasets for CPD.

		Number of European-ancestry subjects per phenotype (trait value)							Demographics					
Dataset	Reference Paper(s)	CPD category 1 (control)	CPD category 2	CPD category 3	CPD category 4	CPD case (category 3 and 4)	CPD cases and controls	Smokers with CPD value	% Female	Mean Age	Median Age	Min Age	Max Age	SD Age
COGEND	[4], [38]	1011	410	274	367	641	**1652**	**2062**	61.3	36.4	37	23	45	5.5
Add Health	[39]	308	149	32	12	44	**352**	**501**	51.5	22.4	22	18	26	1.6
BoMa-aff-bpd		35	95	46	63	109	**144**	**239**	51.0	44.2	43	18	74	12.0
BoMa-aff-mdd		54	108	43	49	92	**146**	**254**	59.8	45.1	45	19	76	11.7
BoMa-scz		28	74	36	54	90	**118**	**192**	43.7	33.7	34	17	65	10.5
CADD	[40], [41]	173	114	36	25	61	**234**	**348**	45.1	18.4	18	17	21	1.7
CPS-II_CPD	[8], [42]	1386	0	363	1095	1458	**2844**	**2844**	59	62.3	62	42	81	5.8
CPS-II_LCA	[42]	624	362	215	246	461	**1085**	**1447**	42	63.8	64	44	79	5.6
ECLIPSE	[43]	137	976	431	347	778	**915**	**1891**	34.0	63.1	64	40	75	7.6
GenMetS	[11]	319	252	53	24	77	**396**	**648**	42.4	47.7	47	30	75	9.9
HPFS_CHD	[44],[45]	191	264	154	90	244	**435**	**699**	0	72.1	73	53	89	7.9
HPFS_KS	[46]	77	65	49	27	76	**153**	**218**	0	65.5	65	54	82	6.5
HPFS_T2D	[47]	309	447	280	201	481	**790**	**1237**	0	71.6	72	53	88	8.0
LHS	[7], [48]	144	549	565	685	1250	**1394**	**1943**	37.7	48.5	49	35	60	6.8
MD Anderson	[15], [49]	250	905	499	637	1136	**1386**	**2291**	43.2	61.6	62	31	92	9.9
MUC12SCS	[50]	96	188	61	76	137	**233**	**421**	31.4	37.0	37	18	68	11.2
MUC12SCTL	[50]	118	84	21	12	33	**151**	**235**	51.5	46.9	48	21	72	14.9
MUCMDCS	[50]	154	285	94	108	202	**356**	**641**	32.5	37	36	18	69	11.3
MUCMDCTL	[50]	503	405	85	59	144	**647**	**1052**	47.4	53.2	58	19	74	14.6
NAG-Aus/BigSib	[51]	592	0	489	248	737	**1329**	**1329**	41	44	43	18	82	9.8
NAG-Finland	[51], [52]	29	133	32	13	45	**74**	**207**	37.8	57.4	56.9	39	91.2	7.6
NCI-EAGLE	[53]	699	1537	498	343	841	**1540**	**3077**	15.9	65.5	66	35	79	8.5
NCI-PLCO	[54]	381	957	643	621	1264	**1645**	**2602**	29.2	64.0	64	55	74	5.0
NHS_BrCa	[55], [56]	305	546	196	163	359	**664**	**1210**	100	70.3	71	56	81	6.3
NHS_CHD	[57]	198	307	153	90	243	**441**	**748**	100	70.9	72	47	81	6.4
NHS_KS	[46]	72	119	37	26	63	**135**	**254**	100	66.6	66	56	81	6.4
NHS_T2D	[47]	481	707	238	220	458	**939**	**1646**	100	69.1	69	48	81	6.5
NYSFS	[58]–[60]	110	110	6	48	54	**164**	**274**	55	18.9	19	16	22	1.9
UK_Phase_II	[61]	563	1608	481	482	963	**1526**	**3134**	39.7	69	70	34	100	8.6
Utah	[7]	63	184	102	137	239	**302**	**486**	41.8	59.3	60	25	86	10.5
UVa-MSTF	[62]	23	96	80	64	144	**167**	**263**	67.3	47.5	48	18.3	82.2	9.0
VA-twin	[63]	620	653	465	650	1115	**1735**	**2388**	30.3	37.8	37	21	62	9.0
WSU	[64]	176	415	155	178	333	**509**	**924**	81.9	53.8	53	19	74	12.1
Yale-UConn	[65]–[67]	216	537	91	68	159	**375**	**912**	40.1	38.4	39	18	71	11.3
**TOTAL**		**10445**	**13641**	**7003**	**7528**	**14531**	**24976**	**38617**						

**Table 2:** Description of contributing datasets for lung cancer.

	Number of European-ancestry subjects			Demographics					
Dataset	Lung cancer cases (smokers)	Lung cancer-free controls (smokers)	Total subjects	% Female	Mean Age	Median Age	Min Age	Max Age	SD Age
CPS-II_LCA	699	748	1447	41.5	63.9	64	44	79	5.6
MD Anderson	1154	1137	2291	43.2	61.6	62	31	92	9.9
NCI-EAGLE	1770	1340	3110	15.8	65.5	66	35	79	8.5
NCI-PLCO	1253	1350	2603	29.2	64.0	64	55	74	5.0
UK_Phase_II	2300	933	3233	39.2	69.4	71	34	100	8.6
WSU	524	406	930	81.9	53.8	53	19	74	12.1
**TOTAL**	**7700**	**5914**	**13614**						

**Table 3:** Description of contributing datasets for COPD.

	Number of European-ancestry subjects			Demographics					
Dataset	COPD cases (smokers)	COPD-free controls (smokers)	Total subjects	% Female	Mean Age	Median Age	Min Age	Max Age	SD Age
CPS-II_CPD	565	2279	2844	59.0	62.3	62	42	81	5.8
CPS-II_LCA	330	1117	1447	41.5	63.9	64	44	79	5.6
ECLIPSE	1719	172	1891	34.0	63.0	64	40	75	7.6
WSU	238	692	930	81.9	53.8	53	19	74	12.1
**TOTAL**	**2614**	**3568**	**6182**						

### Traits for analysis

The traits examined were smoking quantity, lung cancer, and COPD. Two smoking traits were derived from measurements of cigarettes smoked per day (CPD): a 4-level categorical trait (CPD≤10, 10<CPD≤20, 20<CPD≤30, and CPD>30) and a dichotomous trait contrasting subjects from the lowest smoking category (CPD≤10: light-smoking “controls”) to those in the two highest categories combined (CPD>20: heavy smoking cases). The dichotomous trait of heavy versus light smoking was our primary trait for analysis. For one study (NAG-Finland), which used different boundaries to record CPD as detailed in the supplemental material, the distribution of CPD was examined to harmonize the phenotypes and select alternative boundaries. The numbers of subjects in each smoking category, total and by study, are given in Table 1. Lung cancer and COPD were analyzed as dichotomous traits. COPD cases were defined to have COPD as determined by post-bronchodilator spirometry as GOLD Stage II or worse (N = 1,719), or self-reported COPD, emphysema or chronic bronchitis.

### SNPs for analysis

In European-ancestry populations, each of the four loci of interest can be represented by various highly correlated SNPs (SNPs having high r^2^ with each other). For each locus, we chose one target SNP for analysis: rs16969968 (locus 1), rs578776 (locus 2), rs588765 (locus 3), and rs12914008 (locus 4); the pairwise correlations between any two of these loci are r^2^<0.5 (Table S1). In samples for which a given target SNP was not available, we chose a highly correlated proxy SNP based on r^2^ computed with Haploview [26] using downloaded HapMap CEU genotype data, Release 23 [27]. Table S2 lists the proxy SNPs used and their r^2^ with the corresponding target SNPs. Figure S1 displays the SNPs for each of the 4 loci in relation to the *CHRNA5-CHRNA3-CHRNB4* cluster.

### Statistical analyses and meta-analysis

To ensure uniform analyses, SAS (SAS Institute, Cary, NC) and R [28] scripts for genetic association analyses were developed centrally and then distributed. The scripts were executed by each participating site, and the results returned to the coordinating group.

In each dataset, associations between the loci and the traits were evaluated using logistic regression. Our primary analysis model coded genotypes additively as the number of copies of the minor allele according to the HapMap CEU reference population. This allele is referred to as the “coded allele” (C) and the major allele is referred to as the “reference allele” (R). To confirm the appropriateness of the additive model, for each locus a 2 degree of freedom model including the additive term and a heterozygote deviation term was evaluated. The analyses of the 4-level CPD trait used generalized logistic regression to obtain separate effect estimates (beta coefficients) for each category with respect to the lowest smoking category as the referent. All these association analyses included sex and age as covariates. In addition, lung cancer and COPD analyses included categorical cigarettes-per-day as an unordered covariate.

Association results from each dataset, including the beta coefficient and standard error, were provided to the coordinating team. Meta-analysis was carried out using PLINK [29] to obtain overall summary odds ratios (ORs) and statistics. The R package rmeta [30] was used to verify results and create plots. There was no evidence of significant heterogeneity across datasets for these analyses (minimum heterogeneity p = 0.21 for dichotomous CPD, 0.07 for lung cancer, 0.24 for COPD; for categorical CPD a nominally significant p was seen only for category 3 and locus 1 (p = 0.007)). Because of varying study designs, ascertainment strategies, and representative SNPs, we nevertheless report results from random effects meta-analyses.

As noted earlier, locus 1 (representing rs16969968) is a highly replicated association finding and furthermore rs16969968 has been shown to have functional effects on the resulting alpha5-containing receptor [19]. Therefore an important question is whether the remaining loci demonstrate additional independent effects on disease risk. Although loci 2, 3 and 4 are not highly correlated with rs16969968, |D'| is high. A high |D'| can correspond to a low r^2^ if the alleles that tend to co-occur on the same haplotype have very different allele frequencies. Previous results in the COGEND data suggest that there may be independent or synergistic effects on nicotine dependence between locus 1 and locus 3 [4], and haplotype analyses in the Utah and LHS samples [7], and in the COGEND and CPS-II-CPD samples [22], also indicate effects of haplotypes containing loci 1, 2 and 3.

To test whether additional loci contribute to dichotomous smoking quantity over and above the effect of rs16969968, we included both locus 1 and each of the other loci in the logistic regression models adjusting for sex and age, with and without a SNP×SNP interaction term. For lung cancer and COPD the models also included categorical cigarettes-per-day as an unordered covariate. These results were then meta-analyzed as described above. The SNP×SNP interaction term was never significant in the meta-analysis (p>0.3), so we report results from the joint models without interactions. To allow comparison between single-SNP and joint results on comparable data, for each locus pair we also repeated the univariate single-SNP meta-analyses on the subset of datasets that had genotypes available at both loci. For dichotomous smoking quantity we also tabulated pair-wise joint genotype by case status counts for locus 1 (rs16969968) versus each of the other three loci across the contributing datasets that had both loci.

### Multiple test correction

Across the four target loci, multiple traits (4), the multiple models (additive and additive+heterozygote deviation), and the 2-SNP joint analyses (3 loci), our study was designed to perform fewer than 80 tests. A conservative Bonferroni correction would result in an uncorrected p-value threshold of 6.25×10^−4^ corresponding to an experiment-wide alpha of 0.05. The results tables report uncorrected p-values which we compared to this threshold to determine statistical significance.

## Results

We calculated allele frequencies within each sample to confirm that the coded allele (minor allele in HapMap CEU) was indeed the minor allele as expected in these European-ancestry subjects. Table S3 shows allele frequencies in each sample for the SNPs used. For each locus, frequencies are similar across studies and proxy SNPs, and similar to the frequencies in the HapMap CEU reference population.

All reported results are based on additive models. The additive model is appropriate because none of the tests for deviation from the additive assumption were significant. For each analysis, the tables and figures report the number of individuals successfully genotyped for the relevant SNP or SNPs.

### Dichotomous CPD, single-SNP analysis

**Figure 1:**
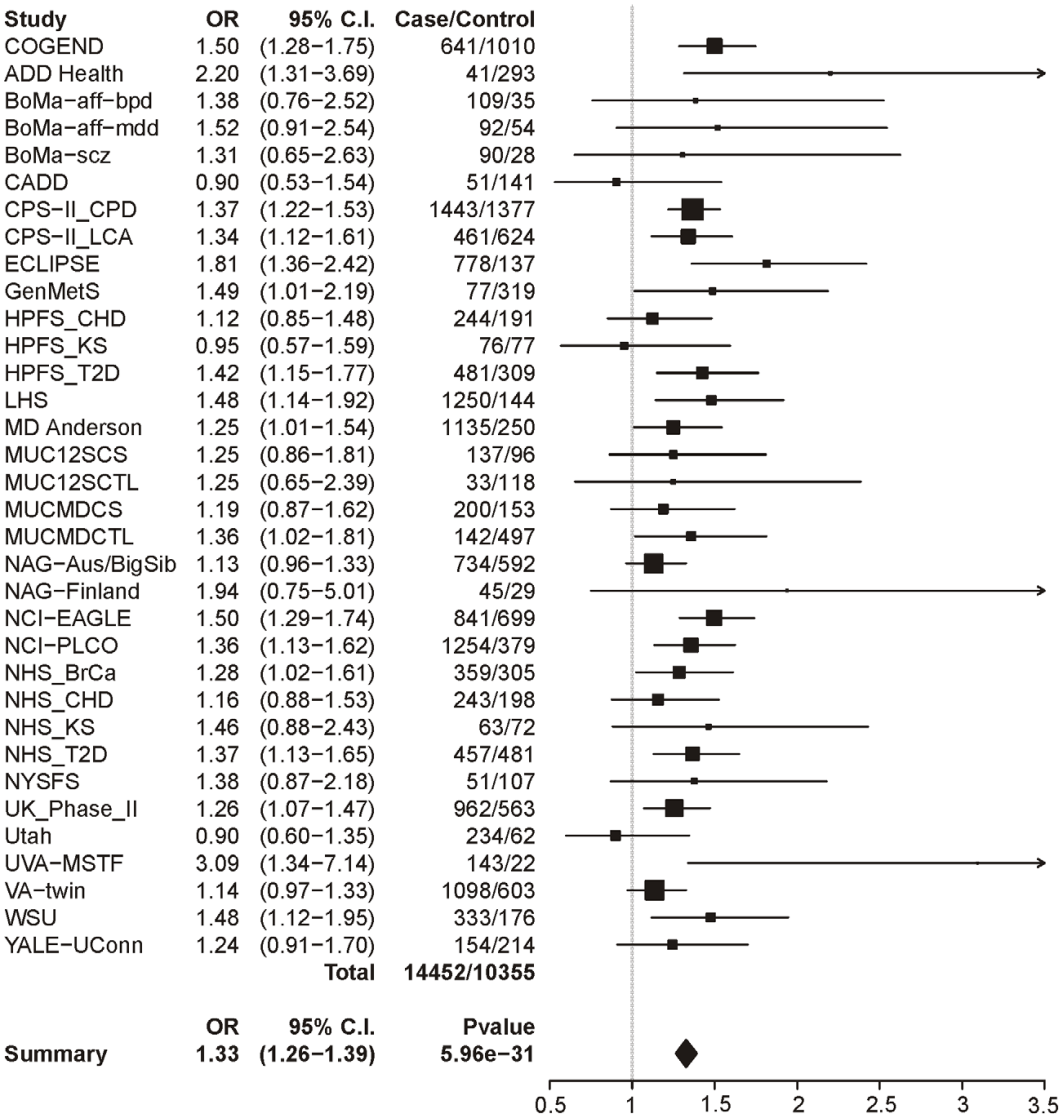
Forest plot for dichotomous CPD at locus 1 (tagging rs16969968). The ORs and 95% CIs are for the effect per allele using additive coding in the logistic regression with age and sex as covariates. The box size indicates the precision of the OR estimate. The case and control totals include only individuals with a genotype call for locus 1. The heterogeneity p-value is 0.21.

Table 4 summarizes the meta-analysis results of dichotomous CPD (heavy/light smoking) in single-SNP analysis. Meta-analysis across all 34 samples clearly shows a highly significant association between dichotomous CPD and locus 1 (tagging rs16969968). Figure 1 displays a forest plot of the summary meta-analysis results for locus 1 (p = 5.96×10^−31^, OR = 1.33, 95% confidence interval (1.26–1.39)), and also the ORs in each contributing dataset.

**Table 4:** Meta-analysis results for dichotomous CPD cases/controls.

	Number of contributing datasets	Number of CPD cases^1^	Number of CPD controls^1^	Summary P-value^2^	Summary OR^2^
Additive test					
Locus 1: rs16969968	34	14452	10355	5.96E-31	1.327
Locus 2: rs578776	32	13391	9524	1.38E-25	0.776
Locus 3: rs588765	33	14101	10149	2.70E-04	0.928
Locus 4: rs12914008	25	11636	8629	4.54E-01	1.045

Logistic regression with sex and age as covariates.

1 Subjects successfully genotyped for the relevant SNP.

2 Random effects meta-analysis.

**Figure 2:**
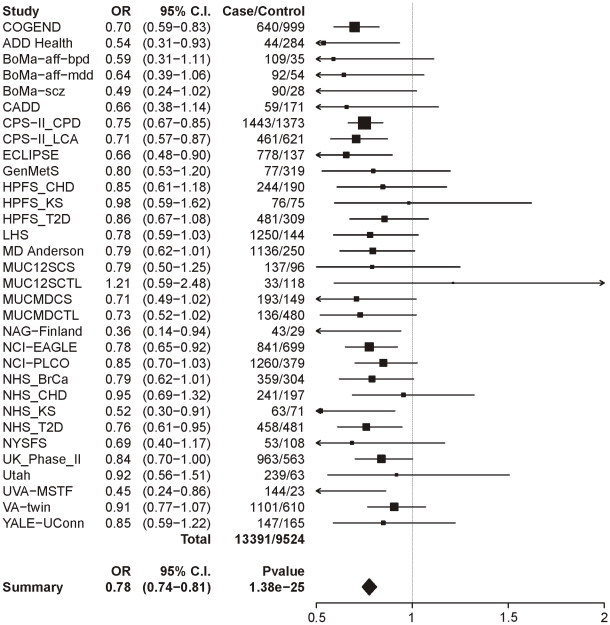
Forest plot for dichotomous CPD at locus 2 (tagging rs578776). The ORs and 95% CIs are for the effect per allele using additive coding in the logistic regression with age and sex as covariates. The heterogeneity p-value is 0.69.

**Figure 3:**
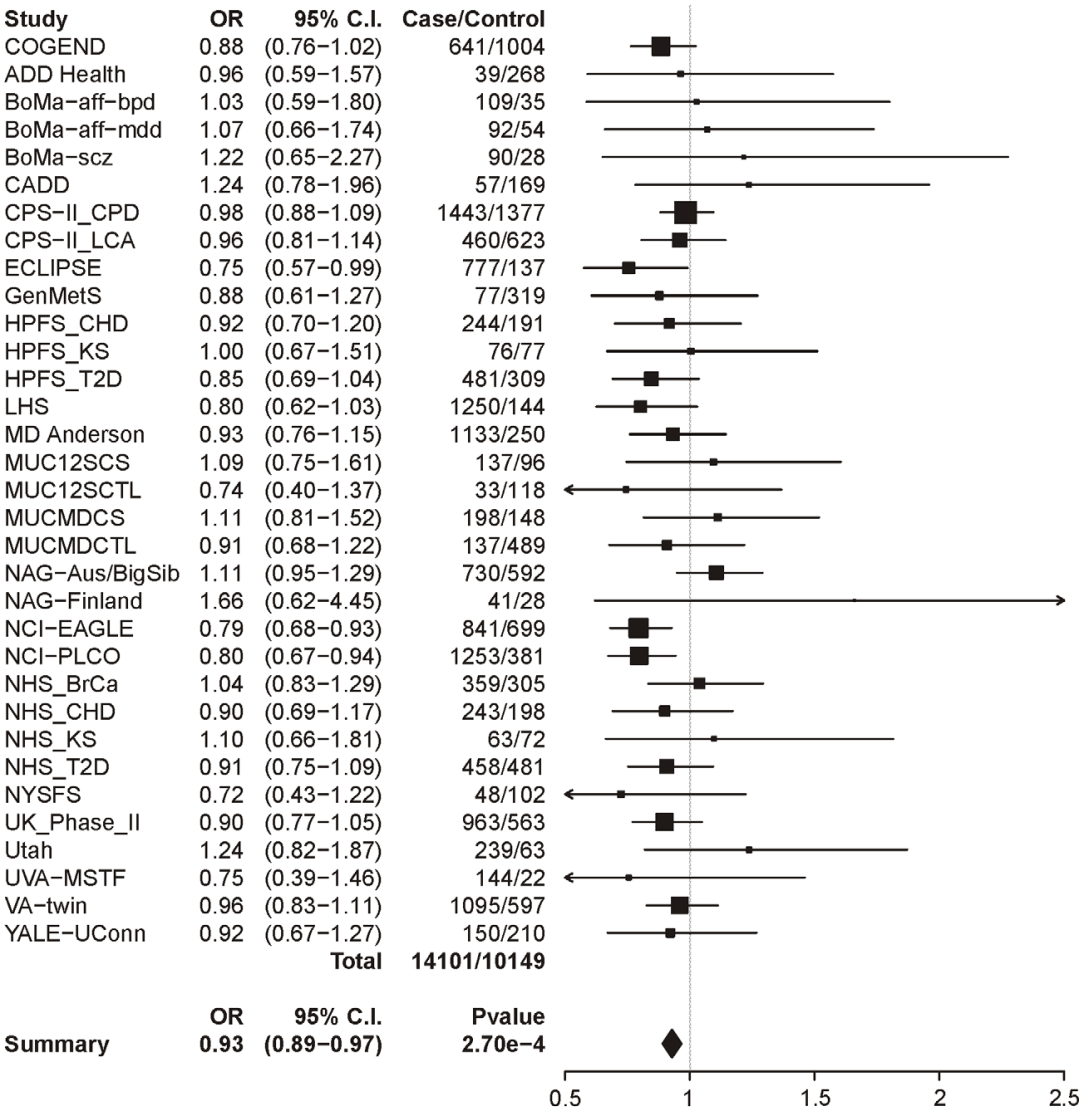
Forest plot for dichotomous CPD at locus 3 (tagging rs588765). The ORs and 95% CIs are for the effect per allele using additive coding in the logistic regression with age and sex as covariates. The heterogeneity p-value is 0.59.

The same analysis of locus 2 (tagging rs578776) yields a meta-analysis p-value of 1.38×10^−25^ and an OR of 0.78 (0.74–0.81), indicating a protective association for the minor allele as has previously been reported (Figure 2). Locus 3 (tagging rs588765) under the same model gives a p-value of 0.00027 and OR of 0.93 (0.89–0.97), which meets our threshold for multiple-test corrected significance but, unlike locus 1 and locus 2, does not surpass genome-wide significance (Figure 3). Locus 4 (tagging rs12914008) does not show a main effect on dichotomous CPD (p = 0.45, OR = 1.05 (0.93–1.17). The forest plot for locus 4 is given in Figure S2.

### Categorical CPD, single-SNP analysis

The categorical CPD analysis, which includes all 4 CPD levels in a generalized logit model, allows us to evaluate genetic effects for each CPD category with respect to the lowest smoking class (CPD≤10). Table 5 shows the results.

**Table 5:** Meta-analysis results for categorical CPD.

	Number of contributing datasets	Number of subjects in the given CPD category^1^	Summary P-value^2^	Summary OR^2^
Locus 1: rs16969968				
Category 1: CPD≤10	34	10355	–	referent
Category 2: 10<CPD≤20	32	13562	3.166E-08	1.149
Category 3: 20<CPD≤30	34	6957	2.121E-12	1.290
Category 4: CPD>30	34	7495	5.470E-40	1.397
Locus 2: rs578776				
Category 1: CPD≤10	32	9524	–	referent
Category 2: 10<CPD≤20	31	13120	2.799E-07	0.883
Category 3: 20<CPD≤30	32	6328	1.353E-12	0.786
Category 4: CPD>30	32	7063	3.387E-20	0.770
Locus 3: rs588765				
Category 1: CPD≤10	33	10149	–	referent
Category 2: 10<CPD≤20	31	13118	1.162E-01	0.967
Category 3: 20<CPD≤30	33	6798	1.142E-02	0.940
Category 4: CPD>30	33	7303	6.251E-05	0.894
Locus 4: rs12914008				
Category 1: CPD≤10	25	8629	–	referent
Category 2: 10<CPD≤20	24	11486	1.215E-01	0.918
Category 3: 20<CPD≤30	25	5483	9.349E-01	1.006
Category 4: CPD>30	25	6153	2.260E-01	1.081

Generalized logistic regression, additive model.

with CPD category 1 as the referent and sex and age as covariates.

1 Subjects successfully genotyped for the relevant SNP.

2 Random effects meta-analysis.

For locus 1 (rs16969968), we see an ordinal effect with increasing CPD; that is, the odds ratio increases from 1.15 to 1.29 to 1.40 for categories 2, 3 and 4, with a corresponding decrease in p-value from 3.17×10^−8^ to 2.12×10^−12^ to 5.47×10^−40^. A similar ordinal effect is seen for locus 2 (rs578776), with the odds ratio decreasing from 0.88 to 0.79 to 0.77. For locus 3 (rs588765) we see an effect only with the highest smoking category (CPD>30). For locus 4 no effect is seen across smoking categories, consistent with the dichotomous CPD results.

### Joint analysis for dichotomous CPD

To dissect the potential distinct effects of these loci on heavy versus light smoking, we carried out meta-analyses of joint SNP models that included sex, age, locus 1 and each of the other loci, coded additively.

In the joint analysis of locus 1 and locus 2, there is suggestive evidence of distinct effects, but the association at locus 2 is no longer genome-wide significant in the presence of locus 1. Both SNPs become less significant compared to their single locus models: in the joint model, locus 1 gives p = 2.15×10^−22^, OR = 1.27 (1.21–1.33) and locus 2 gives p = 4.50×10^−7^, OR = 0.87 (0.83–0.92). When each SNP is placed individually in the model and meta-analyzed across the 32 datasets that provided data for both loci, locus 1 gives p = 1.41×10^−32^, OR = 1.34 while locus 2 gives p = 1.38×10^−25^, OR = 0.76. The risk-increasing alleles at locus 1 (C) and locus 2 (R) are positively correlated, even though the minor alleles are negatively correlated.

In joint analysis of locus 1 and locus 3, locus 1 (rs16969968) yields a p-value of 3.52×10^−36^, OR = 1.47 (1.38–1.56); locus 3 (rs588765) gives p = 6.03×10^−9^, OR = 1.17 (1.11–1.23). Thus locus 3 attains genome-wide significance (p<5×10^−8^) after adjusting for the effect of locus 1. Note that adjusting for locus 1 changes the direction of effect for locus 3 (OR>1) compared to the single-SNP results. In the 33 datasets that have both loci genotyped, we obtain p = 5.39×10^−29^, OR = 1.32 for locus 1 alone, and p = 0.00027, OR = 0.93 (0.89–0.97) for locus 3 alone. The evidence for association in the joint model is stronger than when each SNP is analyzed alone. In fact, when locus 1 is not taken into account, the effect of locus 3 is potentially masked, and the effect of the minor allele is in an opposite direction (protective versus risk).

To further examine these interesting results for locus 1 and locus 3, we show the number of heavy and light smokers in each joint genotype class, and corresponding odds ratios using the genotype that is homozygous for both reference (major) alleles as the reference group (Table 6). The reference alleles (major in HapMap CEU) are labeled “R” and the coded alleles (minor in HapMap CEU) are labeled “C”.

**Table 6:** Joint genotype table for locus 1 versus locus 3 in CPD cases (heavy smokers) and controls (light smokers).

	Locus 3: rs588765 or correlates					
	RR		RC		CC	
Locus 1: rs16969968 or correlates	N cases/N controls^1^	Odds ratio (95% CI)^2^	N cases/N controls^1^	Odds ratio (95% CI)^2^	N cases/N controls^1^	Odds ratio (95% CI)^2^
**RR**	605/672	1.00 (ref)	2317/2237	1.15 (1.02–1.30)	2259/1783	1.41 (1.24–1.60)
**RC**	2302/1757	1.46 (1.28–1.65)	4295/2760	1.73 (1.53–1.95)	37/34	1.21 (0.75–1.95)
**CC**	2185/1073	2.26 (1.98–2.58)	48/28	1.90 (1.18–3.07)	2/1	2.22 (0.20–24.56)

R = reference allele.

C = coded allele.

1 The number of CPD cases and CPD controls with the specified two-locus genotype combination.

2 The odds ratio and 95% CI when the reference joint genotype is RR at locus 1 and RR at locus 3.

The first important observation is that there are very few subjects in certain cells, namely the cells corresponding to RC/CC at locus 1/locus 3, CC/RC, and CC at both loci. This table therefore reveals that the risk alleles at locus 1 (C) and locus 3 (C) are negatively correlated, and explains why the effect of rs588765 is seen only after adjusting for rs16969968. This pattern also reflects the high |D'| between the loci.

The second observation is that for the remaining, well populated cells, the coded allele at locus 3 increases risk on the background of a fixed genotype at locus 1 (e.g. row 1 of the table, corresponding to the stratum of RR homozygotes at locus 1). Similarly, for a fixed genotype at locus 3, the coded allele at locus 1 increases risk (e.g. column 1 of the table, corresponding to the stratum of RR homozygotes at locus 3). Thus for each locus, the effect seen in the joint, 2-SNP logistic regression is confirmed in the most informative stratum at the other locus.

For locus 1 and locus 4 in the joint model, locus 1 gives p = 1.01×10^−38^, OR = 1.35 (1.29–1.41) and locus 4 gives p = 5.55×10^−3^, OR = 1.17 (1.05–1.31). While the effect for locus 4 is stronger than was seen in single-SNP analysis, it does not meet our multiple test threshold for significance. In single-SNP analysis of the 25 datasets that have genotypes at both loci, locus 1 alone gives p = 7.56×10^−35^, OR = 1.33; locus 4 is non-significant (p = 0.45, OR = 1.05).

### Lung cancer controlled for CPD

**Figure 4:**
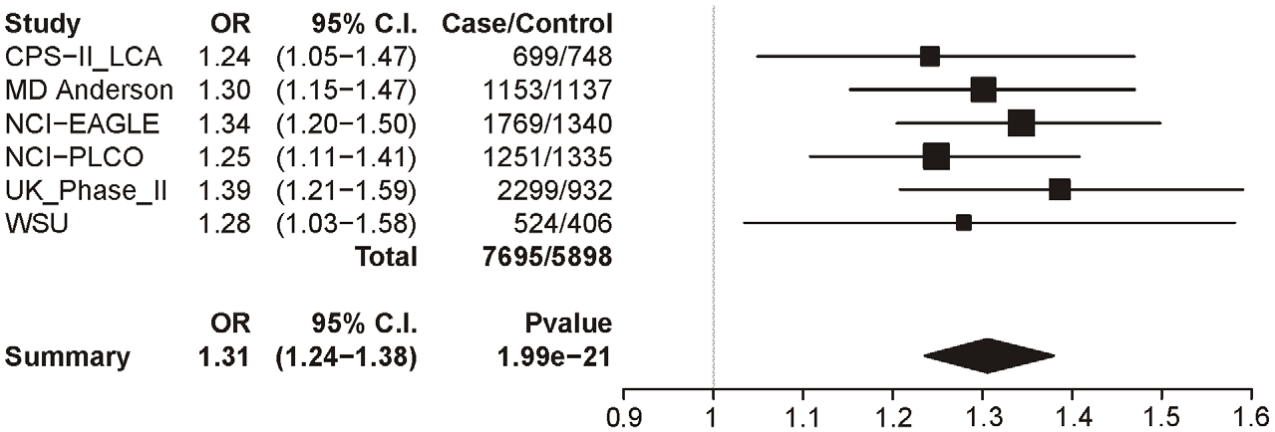
Forest plot for lung cancer at locus 1 (tagging rs16969968). The ORs and 95% CIs are for the effect per allele using additive coding in the logistic regression with age, sex and categorical CPD as covariates. The heterogeneity p-value is 0.86.

In Table 7 we report the single-SNP meta-analysis results for the six lung cancer datasets; recall that all subjects were smokers, and sex, age and categorical CPD were included as covariates. As with the CPD traits, locus 1 (rs16969968) shows highly significant evidence for association with lung cancer (p = 1.99×10^−21^). The summary odds ratio of 1.31 (1.24–1.38) closely matches the dichotomous CPD odds ratio of 1.33 (1.26–1.39). Figure 4 shows the association results for locus 1 by dataset and the overall meta-analysis results.

**Table 7:** Meta-analysis results for lung cancer.

	N (number of contributing datasets)	Number of lung cancer cases^1^	Number of lung-cancer-free controls^1^	Summary P-value^2^	Summary OR^2^
Additive test					
Locus 1: rs16969968	6	7695	5898	1.987E-21	1.306
Locus 2: rs578776	5	7174	5500	9.742E-10	0.818
Locus 3: rs588765	5	7171	5491	4.008E-04	0.904
Locus 4: rs12914008	5	7170	5478	1.941E-01	1.140

Logistic regression with sex, age and categorical CPD as covariates.

1 Subjects successfully genotyped for the relevant SNP.

2 Random effects meta-analysis.

**Figure 5:**
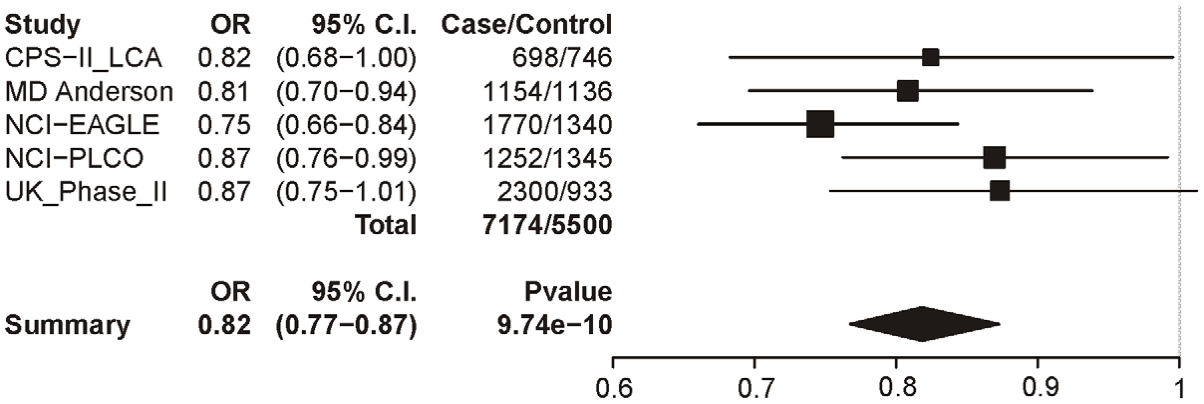
Forest plot for lung cancer at locus 2 (tagging rs578776). The ORs and 95% CIs are for the effect per allele using additive coding in the logistic regression with age, sex and categorical CPD as covariates. The heterogeneity p-value is 0.44.

**Figure 6:**
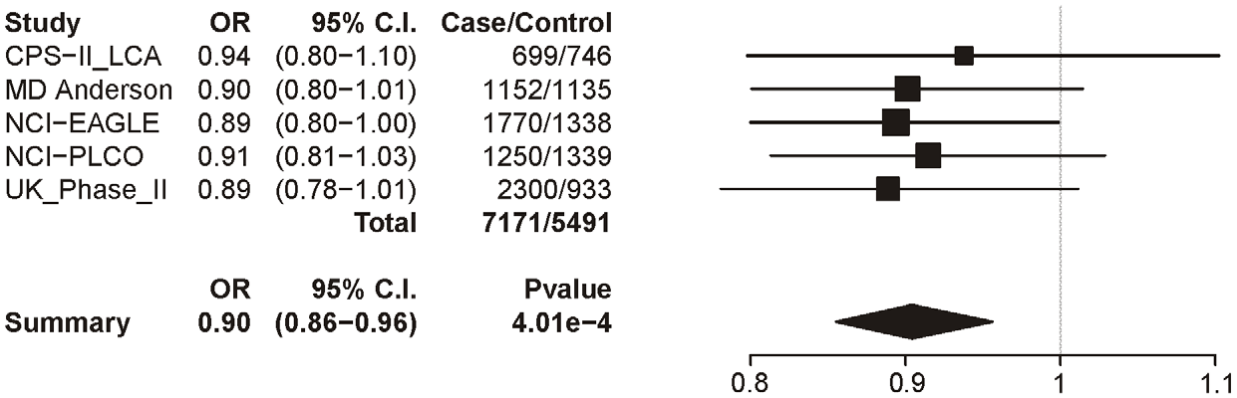
Forest plot for lung cancer at locus 3 (tagging rs588765). The ORs and 95% CIs are for the effect per allele using additive coding in the logistic regression with age, sex and categorical CPD as covariates. The heterogeneity p-value is 0.99.

Locus 2 (rs578776) also shows evidence of association with lung cancer in single-SNP analysis (p = 9.74×10^−10^; OR = 0.82 (0.77–0.87)) (Figure 5). Locus 3 results in a p-value of 0.0004 (OR = 0.90 (0.86–0.96)) (Figure 6); as with categorical CPD, this meets our multiple-test-corrected threshold but is not genome-wide significant. Locus 4 shows no evidence for association with lung cancer; the forest plot is given in Figure S3.

### Joint analyses for lung cancer controlled for CPD

Similar to our analyses of categorical CPD, we carried out joint analyses of locus 1 with each of the other 3 loci, with covariates for sex, age and dummy-coded CPD. After adjusting for the effect of locus 1, none of the other loci reached our multiple-test-corrected significance threshold.

For locus 1 and locus 2 jointly in the model, locus 1 gave p = 2.68×10^−13^, OR = 1.26 (1.19–1.34) and locus 2 gave p = 0.012, OR = 0.91 (0.85–0.98). In joint analysis of locus 1 and locus 3, locus 1 yields p = 2.24×10^−19^, OR = 1.39 (1.30–1.50) and locus 3 gives p = 0.0050, OR = 1.11 (1.03–1.19), showing the same change from protective to risk for the minor allele as was observed in the dichotomous CPD analysis. Finally, in the last pairing, locus 1 gives p = 2.66×10^−22^ OR = 1.33 (1.26–1.41) and locus 4 gives p = 0.028, OR = 1.26 (1.02–1.55).

### COPD controlled for CPD

**Figure 7:**
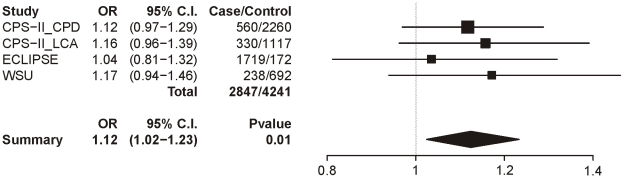
Forest plot for COPD at locus 1 (tagging rs16969968). The ORs and 95% CIs are for the effect per allele using additive coding in the logistic regression with age, sex and categorical CPD as covariates. The heterogeneity p-value is 0.88.

Table 8 summarizes the meta-analysis results for the 3 datasets with the COPD trait; as with lung cancer, all subjects were smokers and sex, age, and categorical CPD were included as covariates. In these analyses, only locus 1 provides even suggestive evidence for association though it does not survive multiple test correction (uncorrected p = 0.01). The locus 1 odds ratio is 1.12 (1.02–1.23), a point estimate lower than that for CPD (1.33) and lung cancer (1.31) (Figure 7).

**Table 8:** Meta-analysis results for COPD.

	N (number of contributing datasets)	Number of COPD cases^1^	Number of COPD controls^1^	Summary P-value^2^	Summary OR^2^
Additive test					
Locus 1: rs16969968	4	2847	4241	1.343E-02	1.124
Locus 2: rs578776	3	2609	3542	3.347E-01	0.934
Locus 3: rs588765	3	2607	3548	1.300E-01	0.922
Locus 4: rs12914008	3	2609	3549	2.364E-01	0.862

Logistic regression with sex, age and categorical CPD as covariates.

1 Subjects successfully genotyped for the relevant SNP.

2 Random effects meta-analysis.

## Discussion

The first goal of this meta-analysis project was to test whether distinct loci in the *CHRNA5-CHRNA3-CHRNB4* gene cluster demonstrate independent effects on smoking behavior (heavy (CPD>20) versus light (CPD≤10) smoking). We selected loci for study based on prior statistical and/or functional evidence for involvement. The second goal was to test whether similar patterns of association are seen across these loci in the smoking-related diseases of lung cancer and COPD. This meta-analysis marks the first large-scale effort to line up association results for these related traits – smoking, lung cancer, and COPD – using a uniform analysis protocol. Our results contribute important new insights about genetic risk for these traits. In particular, we demonstrate strong evidence that smoking behavior is influenced by multiple distinct loci in this region, including two loci that are associated with relevant biological effects in functional studies.

First, our results show that locus 1, representing the *CHRNA5* amino acid change rs16969968 and correlates, demonstrates highly significant association with smoking behavior (OR = 1.33, p = 5.96×10^−31^). Our strong evidence for the involvement of locus 1 with smoking across these samples marks the robustness of its genetic effect. The contributing datasets for the smoking analyses range from samples ascertained for nicotine dependence, lung cancer, or COPD, to adolescent samples, to populations ascertained for a variety of diseases including schizophrenia, alcohol or other substance dependence, breast cancer, type 2 diabetes, and heart disease. This meta-analysis represents a very diverse group, and yet the association between rs16969968 and smoking behavior is consistent.

The second, and novel, finding from this meta-analysis is the evidence for an additional, distinct, locus in this region that is associated with heavy/light smoking and is genome-wide significant. We demonstrated that locus 3, representing rs588765 and correlates, attains a p-value of p = 6.03×10^−9^ (OR = 1.17) when we adjust for locus 1 in a logistic regression model. It is notable that the association between locus 3 and CPD is not as apparent in the single-SNP analysis that does not control for locus 1 (e.g. meta-analysis p = 0.0003, OR = 0.93, which does not reach genome-wide significance). The negative correlation between the risk alleles at locus 1 and locus 3 (r = −0.64) masks the effect at the latter locus in single-SNP analysis, a phenomenon known as suppression [31], [32]. The association evidence for both SNPs is strengthened in the joint analysis, with a reversal of the direction of effect for locus 3. This evidence of statistically independent association for locus 3 with smoking in our analysis is compelling given that these SNPs have also been implicated in altered mRNA levels for *CHRNA5* in brain and lung tissue from European-ancestry subjects [21], [22], [24]. Thus, both statistical and functional evidence indicate that at least one SNP correlated with *CHRNA5* mRNA levels is involved in risk, and highlight locus 3 as an important group of SNPs for further investigation.

A third observation from this study is that locus 2 (rs578776 and correlates) shows evidence for involvement in heavy/light smoking. Locus 2 is genome-wide significant in the single-SNP analysis of dichotomous CPD without adjustment for locus 1, with the minor allele elevated in controls (meta-analysis p = 1.38×10^−25^, OR = 0.78). However the association is much weaker (p = 4.50×10^−7^, OR = 0.87) in the joint logistic regression model that includes locus 1 and locus 2. One interpretation is that part of the single-SNP association at locus 2 is driven by the effect of locus 1 (perhaps related to the high |D'|). Nevertheless, there is evidence for residual signal at locus 2.

We tested a fourth locus representing rs12914008, a relatively uncommon (MAF ∼5%) non-synonymous SNP in *CHRNB4* that has previously shown suggestive evidence for association in European-Americans [4]. In both the univariate analysis and the joint analysis with locus 1, locus 4 is not associated with smoking behavior after multiple test correction. Because of the low allele frequency of this variant, the power to detect an effect is lower than for the other three loci.

This meta-analysis therefore highlights locus 1, locus 2, and locus 3, and indicates dependencies in their effects on risk for heavy smoking. Haplotypes based on these three loci have been described [7], [22] and are seen in HapMap CEU, where the observed haplotype patterns for rs16969968 (locus 1), rs578776 (locus 2), and rs588765 (locus 3) are: A-G-C (frequency 0.425), G-G-T (0.333), G-A-C (0.207), G-A-T (0.035). Only four of the eight possible haplotypes are observed. This is consistent with the correlation structure between the loci. Locus 2 and locus 3 have low correlation with each other (e.g. r^2^ = 0.07 between rs578776 and rs588765 in HapMap CEU release 23); however their correlation sharply increases when locus 1 is taken into account (e.g. in GG homozygotes at rs16969968, r^2^ = 0.74 in HapMap CEU).

Our association results together with the correlation patterns of these three loci suggest that future haplotype or diplotype analyses across large datasets could clarify the relative contributions of these loci. Our evidence that multiple distinct genetic loci affect smoking quantity is consistent with previous reports of risk and protective haplotypes for nicotine dependence in the Utah and LHS samples [7], and in the COGEND and CPS-II-CPD samples [22]. The Utah/LHS study haplotype included 5 SNPs: two that represent locus 1 (rs16969968 and rs1051730), two that represent locus 2 (rs569207 and rs578776), and one that represents locus 3 (rs680244). The COGEND and CPS-II-CPD haplotype analyses included up to 3 loci, one each for locus 1, 2 and 3. Across all these published studies, the high-risk haplotype carries the risk allele at rs16969968 (locus 1); because of the high |D'| between loci, only one haplotype carries that allele. Among the remaining haplotypes, a low risk haplotype is obtained when the minor allele at locus 2 or the major allele at locus 3, or both, is paired with the non-risk allele at rs16969968.

Taken together, our meta-analysis results argue strongly for the existence of at least two statistically distinct loci in this region that affect risk for heavy smoking. In particular, both locus 1 and locus 3, which have known functional effects, are genome-wide significant in joint, mutually-adjusted analysis. The minor allele at locus 3 shifts from a marginally significant protective factor when considered alone to a robust risk factor when considered in combination with locus 1. The statistical evidence and negatively correlated alleles at locus 1 and locus 3 are consistent with at least two mechanistic models: distinct effects of two loci where the minor allele at each locus increases risk across a constant background at the other locus, or a haplotype dose effect where alleles at the two loci act in concert on the same haplotype strand. In the latter model, the minor-major and major-minor haplotypes each increase risk relative to the major-major haplotype, as can be seen in Table 6 once it is recognized that the rarity of the minor-minor haplotype implies that the double-heterozygote cell essentially represents the minor-major and major-minor diplotype. It is also possible that multiple rare variants underlie these findings, as has been suggested in general for disease associations with common SNPs [33]. It remains possible that these associations with locus 1, locus 2 and locus 3 are reflecting correlation with yet another underlying, untyped variant that alone explains the altered biology leading to risk. However, biological involvement of multiple loci appears more likely given that two of these loci represent two distinct, relevant functional consequences: namely, locus 1 (the amino acid change at rs16969968) is associated with altered receptor response to a nicotine agonist *in vitro* [19], and locus 3 (rs588765 and correlates) is associated with altered mRNA levels of *CHRNA5* in brain and lung tissue [22], [24]. Further investigation via resequencing, biological/functional assays, and animal models is needed to dissect the causal biology that underlies the statistical evidence.

An important open question is the degree to which the associations between chr15q25 variants and lung cancer are due to their effects on smoking. When comparing smoking and lung cancer single-SNP results, the patterns of association (odds ratios and directions of effect) were similar across the loci studied. Locus 1 is associated with lung cancer even when controlling for amount smoked per day (p = 1.99×10^−21^, OR = 1.31). This result suggests possible direct genetic effects of locus 1 on this cancer, at least in the presence of smoking. However, CPD is not a sufficient proxy for carcinogen exposure [34], and in never-smokers there is a lack of association between locus 1 and lung cancer [35]–[37], so it is possible that more refined adjustment for smoking will reduce or abolish this association.

For lung cancer, after controlling for categorical CPD and effects of locus 1, we were not able to definitively demonstrate association at either locus 2 or locus 3 after correction for multiple tests. For the mutually adjusted analysis of locus 1 and locus 3 for lung cancer, we observed the same change in the direction for the locus 3 odds ratio that we observed in the joint-SNP analysis of smoking. However, unlike what was seen for smoking, for lung cancer the magnitude (and significance) of the effects did not increase. There are several possible reasons for this, including: chance, the smaller sample size for lung cancer, or qualitative differences in the relationship between these loci and smoking behavior versus the relationship between these loci and lung cancer (after adjusting for smoking quantity). This highlights the challenges posed when attempting to dissect the contributions of multiple loci of modest effect on complex, correlated traits. Further studies, and larger sample sizes, are needed.

For COPD, when controlling for cigarettes-per-day we did not find evidence for association with any of the loci after correction for multiple tests. For locus 1, the odds ratio of 1.12 (1.01–1.23) is lower than for smoking and lung cancer. The COPD analyses were based on smaller samples than those available for CPD or for lung cancer.

Very recently, three other large smoking genetics consortia published their meta-analysis findings that confirm locus 1 (representing not only rs16969918 but also rs1051730 and other SNPs) as the locus most associated with smoking quantity, genome-wide [12]–[14]. All three studies used linear regression to test for association with either quantitative CPD value [14] or categorical CPD (1–10, 11–20, 21–30, and 31+) [12], [13]. Those consortia also report results from conditional analyses in which a locus 1 SNP was included as a covariate, paralleling our joint analyses.

In contrast to our novel finding in CGASP of genome-wide significance for locus 3 when analyzed jointly with locus 1, none of the other consortia report strong evidence for locus 3 when paired with locus 1. In the Oxford-GSK study [13], imputation using 1000 Genomes data detected the most significant single-SNP association for CPD at the locus 1 SNP rs55853698 (r^2^>0.96 with rs16969968). After conditioning on rs55853698, the strongest residual signal was detected at a locus 2 SNP, rs6495308 (p = 3.96×10^−5^; r^2^ = 0.825 with rs578776 in HapMap CEU); they do not report the association result for rs588765 in the conditioned analysis, although it must have been less significant than 3.96×10^−5^. In their single-SNP analysis, rs6495308 (locus 2) gave a p-value of 2.2×10^−10^. Their results for locus 2 are therefore consistent with our observation that in joint analysis of locus 1 and locus 2, the significance at locus 2 is reduced compared to the single-SNP analysis. They do not report on whether the evidence for locus 1 and locus 3 strengthens in the joint analysis compared to single-SNP analysis, as we observed in the CGASP datasets. They do note that there is no obvious residual association with a third SNP after conditioning on either the pairing of locus 1 (rs16969968) and locus 3 (rs588765), or the pairing of locus 1 (rs55853698) and locus 2 (rs6495308). That result is consistent with the correlation and haplotype structure of these three loci discussed previously.

In the ENGAGE study [12], conditioning on the locus 1 SNP rs1051730 identified residual evidence at rs2869046 (p = 4.8×10^−5^) and rs2036534 (p = 9.1×10^−5^), neither of which is genome-wide significant. Rs2036534 tags locus 2 (r^2^ = 0.74 with rs578776 in HapMap CEU) while rs2869046 is only weakly correlated with locus 3 (r^2^ = 0.46).

In TAG [14], the conditional analyses indicated residual association at rs684513 (p = 6.3×10^−9^), rs9788682 (p = 1.06×10^−8^), and rs7163730 (p = 1.22×10^−8^), which attain genome-wide significance. These SNPs are each correlated with locus 2, and much less correlated with locus 3 (r^2^ = 0.7, 0.55 and 0.56 respectively with rs578776 in HapMap CEU; r^2^<0.11 with rs588765). It is possible that differences in samples, phenotype definitions, or analysis methods may be contributing to the differences between our strong findings for locus 3 and the three other consortium reports. To further understand the genetic contributions in this region, more work is needed, and not only statistical evidence but also biological evidence will be important.

In summary, our meta-analysis demonstrates significant, robust association of locus 1, representing the non-synonymous *CHRNA5* SNP rs16969968 as well as rs1051730 and rs55853698, with smoking heaviness across very diverse datasets. Our study also demonstrates strong evidence that at least one additional distinct locus in this region affects risk for heavy smoking. In particular, we have identified for the first time that locus 3 – representing the *CHRNA5* expression-associated SNPs rs588765 and correlates – surpasses GWAS-level significance for association with heavy smoking in European-ancestry subjects; this effect is detectable after adjusting for the effect of rs16969968. This new result for locus 3 raises the corresponding SNPs (rs588765 and correlates) to the level of interest already accorded to the two loci which have previously been detected at GWAS-level significance in single-SNP analyses: locus 1 (rs16969968 and correlates) and locus 2 (rs578776 and correlates). Our result also has implications for all genetic association studies, as it illustrates that joint analysis of SNPs is an important tool for identifying genome-wide significant effects that, soberingly, may be obscured in single SNP analyses.

Our study used multiple highly correlated SNPs to represent each of the 4 tested loci, depending on availability in each dataset, and all subjects were of European ancestry. Hence this study is not designed to determine which SNP(s), among the highly correlated SNPs for each locus, are most likely to be biologically involved. Future work, involving large-scale meta-analysis of other populations (e.g. Asian or African ancestry) to capitalize on LD differences between populations, comprehensive functional annotation of genetic variants, DNA re-sequencing and variant discovery, and functional and animal studies may help narrow down these large sets of correlated SNPs to the most promising causal alleles.

## Supporting Information

Figure S1 The *CHRNA5-CHRNA3-CHRNB4* region containing the target SNPs rs16969968 (locus 1), rs578776 (locus 2) rs588765 (locus 3), and rs12914008 (locus 4). The SNPs used in this study to represent each locus are drawn with dotted lines connecting them to each other.
(1.09 MB TIF)

Figure S2 Forest plot for dichotomous CPD and locus 4.
(0.38 MB TIF)

Figure S3 Forest plot for lung cancer and locus 4.
(0.30 MB TIF)

Table S1 Correlation (r-squared) between the four target SNPs representing loci 1, 2, 3, and 4 (HapMap CEU Release 23).
(0.05 MB DOC)

Table S2 Correlation (r-squared) between the target SNPs and their proxies (HapMap CEU Release 23).
(0.06 MB DOC)

Table S3 Genotyped SNPs and overall allele frequencies, by sample dataset.
(0.10 MB DOC)

Text S1 Descriptions of contributing datasets. Numbered according to appearance in Table 1 and Figure 1.
(0.17 MB DOC)
