# Supplementary figures and images for: Multiple Independent Loci at Chromosome 15q25.1 Affect Smoking Quantity: a Meta-Analysis and Comparison with Lung Cancer and COPD
Source: PLoS Genet. 2010 Aug 5;6(8):e1001053. doi: 10.1371/journal.pgen.1001053 (PMC2916847; doi:10.1371/journal.pgen.1001053)

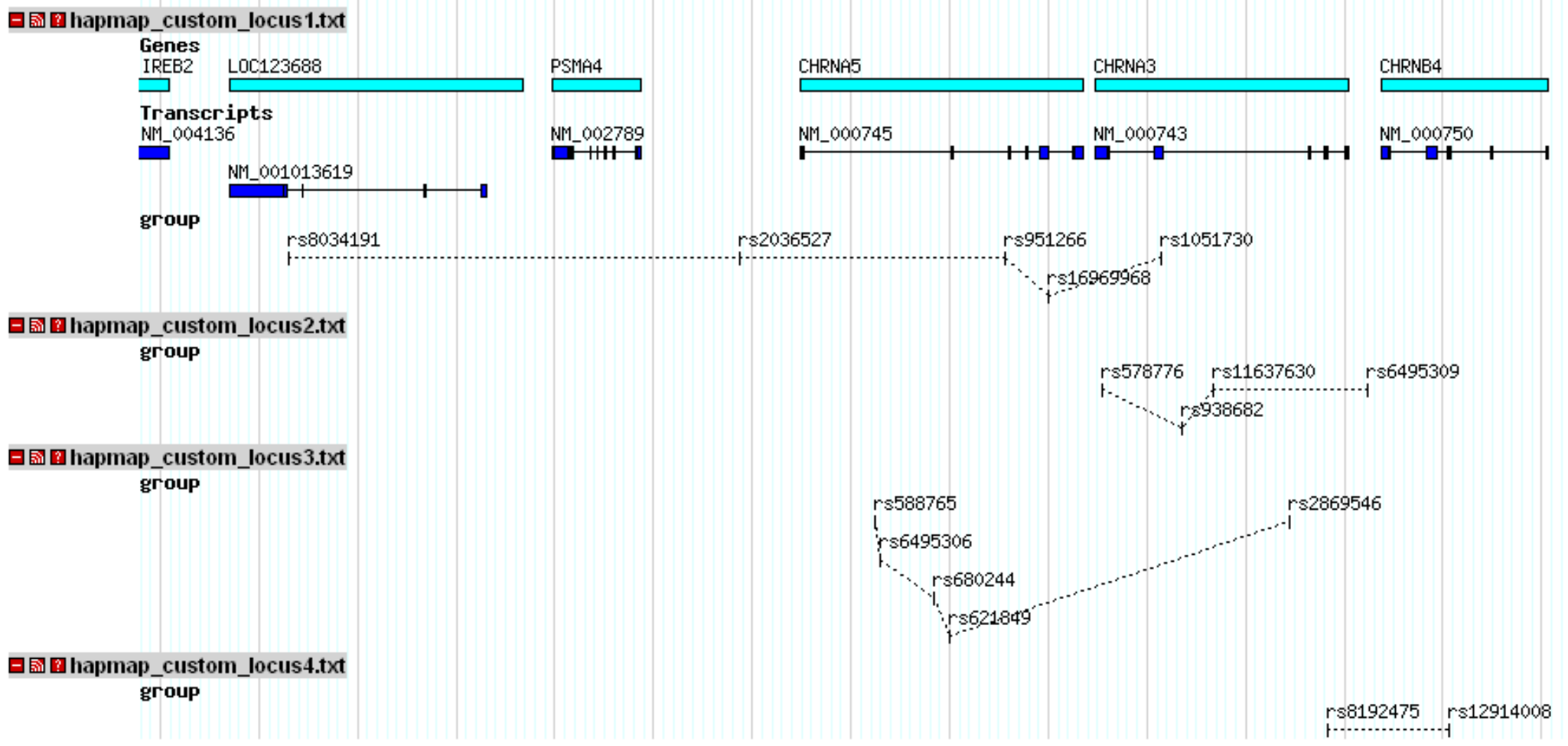

Supplement: Figure S1 — The CHRNA5-CHRNA3-CHRNB4 region containing the target SNPs rs16969968 (locus 1), rs578776 (locus 2) rs588765 (locus 3), and rs12914008 (locus 4). The SNPs used in this study to represent each locus are drawn with dotted lines connecting them to each other. (1.09 MB TIF) [file pgen.1001053.s001.tif]

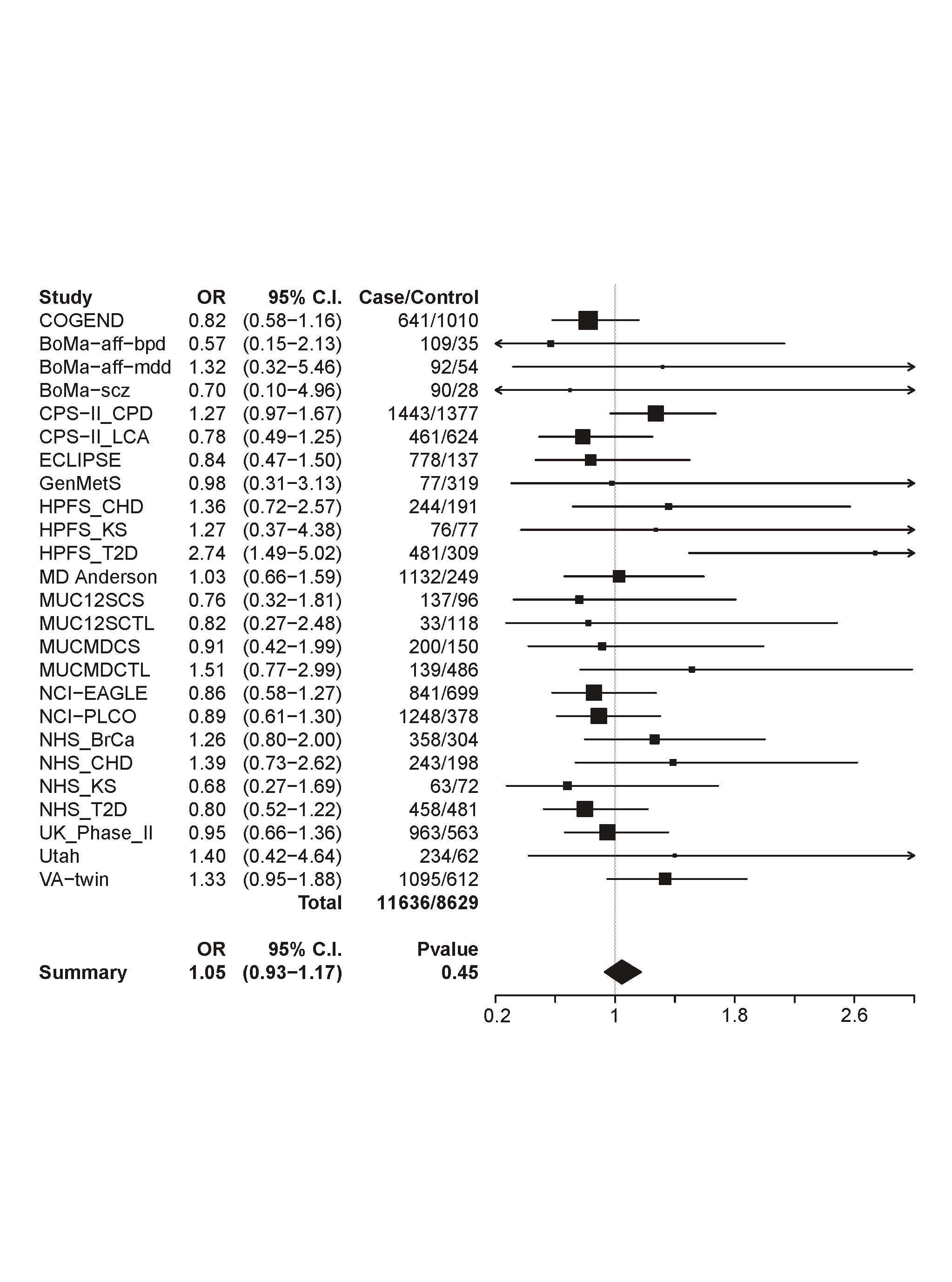

Supplement: Figure S2 — Forest plot for dichotomous CPD and locus 4. (0.38 MB TIF) [file pgen.1001053.s002.tif]

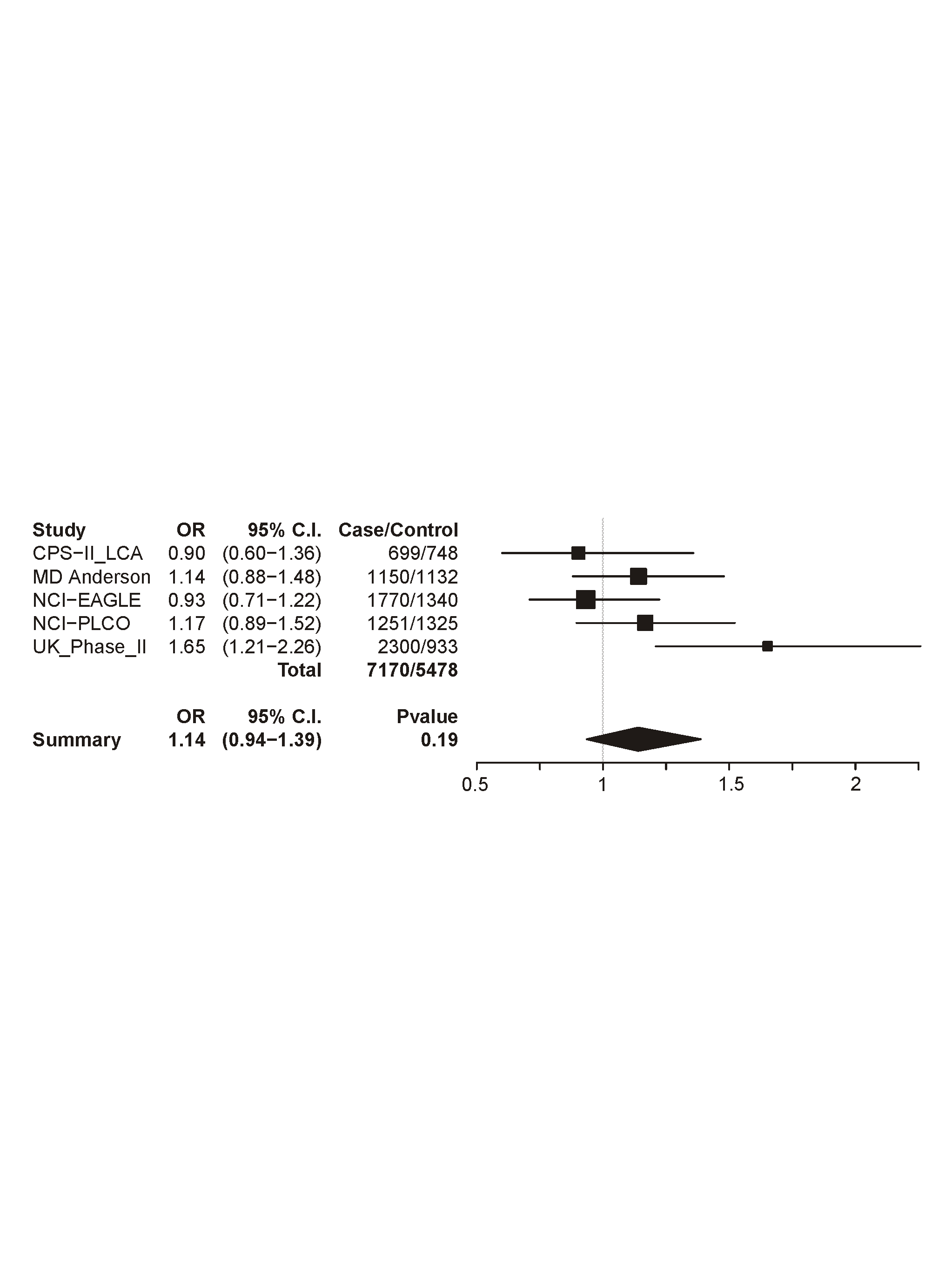

Supplement: Figure S3 — Forest plot for lung cancer and locus 4. (0.30 MB TIF) [file pgen.1001053.s003.tif]
