# Supplementary material for: Multiple Independent Loci at Chromosome 15q25.1 Affect Smoking Quantity: a Meta-Analysis and Comparison with Lung Cancer and COPD
Source: PLoS Genet. 2010 Aug 5;6(8):e1001053. doi: 10.1371/journal.pgen.1001053 (PMC2916847; doi:10.1371/journal.pgen.1001053)
Supplement: Table S1 — Correlation (r-squared) between the four target SNPs representing loci 1, 2, 3, and 4 (HapMap CEU Release 23). (0.05 MB DOC) [file pgen.1001053.s004.doc]

Supporting Table S1. Correlation (r^2^) between the four target SNPs representing

loci 1, 2, 3 and 4 (HapMap CEU Release 23)

| Locus |  | 2 | 3 | 4 |
| --- | --- | --- | --- | --- |
|  | Target SNP | rs578776 | rs588765 | rs12914008 |
| 1 | rs16969968 | 0.240 | 0.407 | 0.026 |
| 2 | rs578776 |  | 0.066 | 0.108 |
| 3 | rs588765 |  |  | 0.035 |
