# Supplementary material for: Multiple Independent Loci at Chromosome 15q25.1 Affect Smoking Quantity: a Meta-Analysis and Comparison with Lung Cancer and COPD
Source: PLoS Genet. 2010 Aug 5;6(8):e1001053. doi: 10.1371/journal.pgen.1001053 (PMC2916847; doi:10.1371/journal.pgen.1001053)
Supplement: Table S2 — Correlation (r-squared) between the target SNPs and their proxies (HapMap CEU Release 23). (0.06 MB DOC) [file pgen.1001053.s005.doc]

Supporting Table S2. Correlation (r^2^) between the target SNPs and their proxies.

| Locus | Target SNP | position (bp) | Proxy SNP | position (bp) | r^2^ |
| --- | --- | --- | --- | --- | --- |
| 1 | rs16969968 | 76669980 | rs8034191 | 76593078 | 0.966 |
|  |  |  | rs2036527 | 76638670 | 0.932 |
|  |  |  | rs951266 | 76665596 | 0.966 |
|  |  |  | rs1051730 | 76681394 | 0.900 |
|  |  |  |  |  |  |
| 2 | rs578776 | 76675455 | rs938682 | 76683602 | 0.826 |
|  |  |  | rs11637630 | 76686774 | 0.825 |
|  |  |  | rs6495309 | 76702300 | 0.784 |
|  |  |  |  |  |  |
| 3 | rs588765 | 76652480 | rs6495306 | 76652948 | 1.000 |
|  |  |  | rs680244 | 76658343 | 1.000 |
|  |  |  | rs621849 | 76659916 | 1.000 |
|  |  |  | rs2869546 | 76694400 | 0.885 |
|  |  |  |  |  |  |
| 4 | rs12914008 | 76710560 | rs8192475 | 76698285 | 0.793 |
