# Supplementary material for: Multiple Independent Loci at Chromosome 15q25.1 Affect Smoking Quantity: a Meta-Analysis and Comparison with Lung Cancer and COPD
Source: PLoS Genet. 2010 Aug 5;6(8):e1001053. doi: 10.1371/journal.pgen.1001053 (PMC2916847; doi:10.1371/journal.pgen.1001053)
Supplement: Table S3 — Genotyped SNPs and overall allele frequencies, by sample dataset. (0.10 MB DOC) [file pgen.1001053.s006.doc]

Supporting Table S3. Genotyped SNPs and overall allele frequencies, by sample dataset.

|  | LOCUS 1 |  |  | LOCUS 2 |  |  | LOCUS 3 |  |  | LOCUS 4 |  |  |
| --- | --- | --- | --- | --- | --- | --- | --- | --- | --- | --- | --- | --- |
| Dataset | locus 1 SNP | HapMap  Alleles R/C^1^ | Coded allele freq^2^ | locus 2 SNP | HapMap  Alleles R/C^1^ | Coded allele freq^2^ | locus 3 SNP | HapMap  Alleles R/C^1^ | Coded allele freq^2^ | locus 4 SNP | HapMap  Alleles R/C^1^ | Coded allele freq^2^ |
| COGEND | rs16969968 | G/A | 0.35 | rs578776 | C/T | 0.274 | rs588765 | C/T | 0.424 | rs12914008 | G/A | 0.045 |
| Add Health | rs16969968 | G/A | 0.3 | rs11637630 | A/G | 0.264 | rs680244 | G/A | 0.425 |  |  |  |
| BoMa-aff-bpd | rs8034191 | T/C | 0.364 | rs578776 | C/T | 0.289 | rs6495306 | A/G | 0.391 | rs8192475 | G/A | 0.04 |
| BoMa-aff-mdd | rs8034191 | T/C | 0.39 | rs578776 | C/T | 0.266 | rs6495306 | A/G | 0.388 | rs8192475 | G/A | 0.037 |
| BoMa-scz | rs8034191 | T/C | 0.359 | rs578776 | C/T | 0.266 | rs6495306 | A/G | 0.422 | rs8192475 | G/A | 0.029 |
| CADD | rs16969968 | G/A | 0.302 | rs11637630 | A/G | 0.237 | rs680244 | G/A | 0.453 |  |  |  |
| CPS-II_CPD | rs16969968 | G/A | 0.35 | rs578776 | C/T | 0.273 | rs588765 | C/T | 0.42 | rs12914008 | G/A | 0.04 |
| CPS-II_LCA | rs8034191 | T/C | 0.368 | rs578776 | C/T | 0.252 | rs621849 | A/G | 0.421 | rs8192475 | G/A | 0.041 |
| ECLIPSE | rs8034191 | T/C | 0.419 | rs578776 | C/T | 0.249 | rs621849 | A/G | 0.385 | rs8192475 | G/A | 0.049 |
| GenMetS | rs8034191 | T/C | 0.347 | rs578776 | C/T | 0.301 | rs621849 | A/G | 0.377 | rs8192475 | G/A | 0.025 |
| HPFS_CHD | rs951266 | C/T | 0.346 | rs938682 | T/C | 0.234 | rs6495306 | A/G | 0.422 | rs8192475 | G/A | 0.044 |
| HPFS_KS | rs8034191 | T/C | 0.349 | rs578776 | C/T | 0.302 | rs6495306 | A/G | 0.401 | rs8192475 | G/A | 0.037 |
| HPFS_T2D | rs951266 | C/T | 0.357 | rs938682 | T/C | 0.236 | rs6495306 | A/G | 0.4 | rs8192475 | G/A | 0.04 |
| LHS | rs16969968 | G/A | 0.389 | rs578776 | C/T | 0.244 | rs680244 | G/A | 0.417 |  |  |  |
| MD Anderson | rs8034191 | T/C | 0.368 | rs6495309 | C/T | 0.193 | rs621849 | A/G | 0.429 | rs8192475 | G/A | 0.053 |
| MUC12SCS | rs8034191 | T/C | 0.367 | rs6495309 | C/T | 0.189 | rs621849 | A/G | 0.429 | rs8192475 | G/A | 0.051 |
| MUC12SCTL | rs8034191 | T/C | 0.355 | rs6495309 | C/T | 0.206 | rs621849 | A/G | 0.428 | rs8192475 | G/A | 0.066 |
| MUCMDCS | rs16969968 | G/A | 0.369 | rs578776 | C/T | 0.246 | rs680244 | G/A | 0.421 | rs12914008 | G/A | 0.037 |
| MUCMDCTL | rs16969968 | G/A | 0.353 | rs578776 | C/T | 0.269 | rs680244 | G/A | 0.422 | rs12914008 | G/A | 0.034 |
| NAG Finland | rs2036527 | C/T | 0.372 | rs578776 | C/T | 0.296 | rs621849 | A/G | 0.359 |  |  |  |
| NAG-Aus/BigSib | rs1051730 | C/T | 0.356 |  |  |  | rs621849 | A/G | 0.426 |  |  |  |
| NCI-EAGLE | rs8034191 | T/C | 0.433 | rs578776 | C/T | 0.257 | rs6495306 | A/G | 0.346 | rs8192475 | G/A | 0.04 |
| NCI-PLCO | rs8034191 | T/C | 0.356 | rs578776 | C/T | 0.266 | rs6495306 | A/G | 0.421 | rs8192475 | G/A | 0.049 |
| NHS_BrCa | rs8034191 | T/C | 0.348 | rs578776 | C/T | 0.272 | rs6495306 | A/G | 0.425 | rs8192475 | G/A | 0.051 |
| NHS_CHD | rs951266 | C/T | 0.352 | rs938682 | T/C | 0.219 | rs6495306 | A/G | 0.428 | rs8192475 | G/A | 0.048 |
| NHS_KS | rs8034191 | T/C | 0.325 | rs578776 | C/T | 0.28 | rs6495306 | A/G | 0.455 | rs8192475 | G/A | 0.059 |
| NHS_T2D | rs951266 | C/T | 0.352 | rs938682 | T/C | 0.241 | rs6495306 | A/G | 0.403 | rs8192475 | G/A | 0.044 |
| NYSFS | rs16969968 | G/A | 0.344 | rs11637630 | A/G | 0.245 | rs680244 | G/A | 0.431 |  |  |  |
| UK_Phase_II | rs16969968 | G/A | 0.363 | rs578776 | C/T | 0.257 | rs588765 | C/T | 0.432 | rs12914008 | G/A | 0.051 |
| Utah | rs16969968 | G/A | 0.381 | rs578776 | C/T | 0.239 | rs680244 | G/A | 0.412 | rs8192475 | G/A | 0.036 |
| UVa-MSTF | rs16969968 | G/A | 0.354 | rs578776 | C/T | 0.288 | rs621849 | A/G | 0.416 |  |  |  |
| VA-twin | rs16969968 | G/A | 0.335 | rs578776 | C/T | 0.275 | rs2869546 | T/C | 0.389 | rs8192475 | G/A | 0.055 |
| WSU-2 | rs16969968 | G/A | 0.383 |  |  |  |  |  |  |  |  |  |
| Yale-UConn | rs16969968 | G/A | 0.349 | rs578776 | C/T | 0.291 | rs621849 | A/G | 0.405 |  |  |  |

^1^ The reference (R) allele is the major allele in HapMap CEU; the coded (C) allele is the minor allele in HapMap CEU.

^2^ Coded allele frequency in all smokers with measured CPD categorical phenotype (values 1, 2, 3 or 4).
