# Supplementary material for: Multiple Independent Loci at Chromosome 15q25.1 Affect Smoking Quantity: a Meta-Analysis and Comparison with Lung Cancer and COPD
Source: PLoS Genet. 2010 Aug 5;6(8):e1001053. doi: 10.1371/journal.pgen.1001053 (PMC2916847; doi:10.1371/journal.pgen.1001053)
Supplement: Text S1 — Descriptions of contributing datasets. Numbered according to appearance in Table 1 and Figure 1. (0.17 MB DOC) [file pgen.1001053.s007.doc]

**Supporting Text S1: Descriptions of contributing datasets. Numbered according to appearance in Table 1 and Figure 1.**

**1. Collaborative Genetic Study of Nicotine Dependence (COGEND)**

The Collaborative Genetic Study of Nicotine Dependence (COGEND) is a United States multi-site project. Subjects were recruited from St. Louis, Detroit, and Minneapolis through community-based telephone screening to determine eligibility for the study. Cases were required to have current Fagerström Test for Nicotine Dependence (FTND) ≥ 4 and controls were required to have a lifetime maximum FTND of 0 or 1, even during the period of heaviest smoking. The number of cigarettes per day (CPD) was assessed for the period of heaviest smoking as well as for current and other time points; the maximum of these values was used to define the CPD trait for analysis.

For this meta-analysis, COGEND contributed a sample of 2062 unrelated European-Americans (641 nicotine dependent cases and 1011 non-dependent smoking controls). All subjects were smokers and reported smoking ≥ 100 cigarettes lifetime. The study obtained informed consent from participants and approval from the appropriate institutional review boards.

DNA was derived from whole blood maintained by the Rutgers University Cell and DNA Repository following stringent quality control and assurance procedures ([www.rucdr.org](http://www.rucdr.org/)). Genotyping of the DNA samples was carried out using Perlegen, Illumina GoldenGate, and Sequenom MassArray iPLEX technology. Cleaning procedures have been detailed^1,2^. Briefly, DNA samples with call rates < 90% were dropped; SNPs were required to pass a call rate threshold of 98%; self-reported race was verified using EIGENSTRAT^3^.

References Cited:

1. Saccone NL, Saccone SF, Hinrichs AL, Stitzel JA, Duan W, Pergadia ML, Agrawal A, Breslau N, Grucza RA, Hatsukami D, Johnson EO, Madden PAF, Swan GE, Wang JC, Goate AM, Rice JP, Bierut LJ. Multiple distinct risk loci for nicotine dependence identified by dense coverage of the complete family of nicotinic receptor subunit (*CHRN*) genes (2009). *American Journal of Medical Genetics Part B: Neuropsychiatric Genetics*. 150B:453-466.

2. Saccone NL, Wang JC, Breslau N, Johnson EO, Hatsukami D, Saccone SF, Grucza RA, Sun L, Duan W, Budde J, Culverhouse RC, Fox L, Hinrichs AL, Steinbach JH, Wu M, Rice JP, Goate AM, Bierut LJ. The *CHRNA5-CHRNA3-CHRNB4* nicotinic receptor subunit gene cluster affects risk for nicotine dependence in African-Americans and in European-Americans (2009). *Cancer Research* 69: 6848-6856.

3. Price AL, Patterson NJ, Plenge RM, et al. Principal components analysis corrects for stratification in genome-wide association studies (2006). *Nat Genet* 38:904-9.

**2.** **The National Longitudinal Study of Adolescent Health (Add Health)**

The National Longitudinal Study of Adolescent Health (Add Health) is a longitudinal study of adolescents in grades 7-12 in the United States during the 1994-1995 school year. A sample of 80 high schools and 52 middle schools was systematically selected to ensure the sample was representative of United States schools with respect to region, urbanicity, school size, school type, and ethnicity. The Add Health cohort has been followed into young adulthood with four in-home interviews (corresponding to Waves I, II, III, IV) <http://www.cpc.unc.edu/projects/addhealth/projects/addhealth>. The most recent interview occurred in 2008, when the sample was aged 24-32. Survey data include respondents’ social, economic, psychological and physical well-being with contextual data on family, neighborhood, community, school, friendships, peer groups, and romantic relationships. The Add Health genetic pairs sample includes pairs of individuals with varying genetic similarity including monozygotic twins, dizygotic twins, full siblings, half siblings, and unrelated siblings who were raised in the same household^1^.

The number of cigarettes-per-day (CPD) was taken from the *Tobacco, Alcohol, Drugs* section of the in-home questionnaire for Waves I, II and III (as Wave IV data are not yet public). CPD was assessed for the period of heaviest smoking (Wave III questionnaire) and from current use for Waves I, II, and III; the maximum of these values was used to define the CPD trait for analyses. A total of 1478 Caucasian non-Hispanic sibling pairs were genotyped from this community sample. For this meta-analysis, Add Health contributed a sample of 862 unrelated, Caucasian, non-Hispanic subjects (self-reported) from the genetic pairs sample (1 randomly extracted individual from each family). Of this subsample, 501 reported smoking. Before the start of the interview, the interviewer described the interview and obtained consent for participation.

DNA was derived from buccal cells collected from the genetic pairs sample. Genomic DNA was preamplified with the method of Zheng^2^. Taqman assays for allelic discrimination (Applied Biosystems, Foster City, CA) were used to determine SNP genotypes. QC performed on the genotyped sample (by sample and by SNP) excluded individuals with less than 50% genotypes (assumed poor quality DNA sample). All SNPs had greater than 95% genotype calling after exclusion of individuals with low quality DNA samples. All genotypes were called by two independent individuals.

References Cited:

1. Harris KM, Halpern CT, Smolen A, Haberstick BC (2006). The National Longitudinal Study of Adolescent Health (Add Health) Twin Data. Twin Research and Human Genetics 9(6): 988-997.
2. Zheng S, Ma X, Buffler PA, Smith MT, Wiencke JK (2001). Whole genome amplification increases the efficiency and validity of buccal cell genotyping in pediatric populations. *Cancer Epidemiol Biomarkers Prev* 10: 697–700.

**3, 4, 5. University of Bonn and University of Mannheim (BoMa-aff-bpd; BoMa-aff-mdd;**

**BoMa-scz)**

The BoMa sample (n=1390) is comprised of three subsamples: patients with a DSM-IV diagnosis of: i) major depression (n=597), ii) bipolar affective disorder (n=313), iii) schizophrenia (n=480) included in GWAS on the above mentioned disorders and originating from larger samples collected for association studies on the respective phenotypes. Patients were recruited from consecutive admissions to the Department of Psychiatry of the University of Bonn, and the Central Institute of Mental Health, Germany and were all of self reported German ancestry/ethnicity. Written informed consent was obtained from all the participants.

Genomic DNA was prepared from whole blood according to standard procedures. Genotyping of the patients was performed using Illumina HumanHap550v3 bead chips. Quality control procedures were followed. DNA samples with call rates <98% were dropped and SNPs were required to pass the following filters: call rate > 98% and minor allele frequency ≥0.01. Self reported ancestry was verified using EIGENSOFT^1^.

References Cited:

1. Price AL, Patterson NJ, Plenge RM, et al. (2006) Principal components analysis corrects for stratification in genome-wide association studies. *Nat Genet* 38:904-9.

**6.** **Family, Twin, and Adoption Studies of the Colorado Center on Antisocial Drug Dependence (CADD)**

A subset of 1075 participants was drawn from the full Colorado Center on Antisocial Drug Dependence (CADD) sample encompassing over 5000 youth, where the inclusion criteria included those assessed between ages 17 and 21. The full CADD sample consists of adolescents from both clinical and community populations^1,2^. Clinical probands were recruited from three treatment facilities in the Denver metropolitan area. The probands were 13-19 years of age at time of assessment and were drawn from individuals with consecutive admissions to the treatment facilities between February 1993 and June 2001. The community-based sample included monozygotic and dizygotic twins and their non-twin siblings drawn from the Colorado Twin Registry. Additional community samples were drawn from two other Colorado community-based family samples: the Colorado Adoption Project (CAP) and the Colorado Adolescent Substance Abuse (ASA) family study^3^. All study participants were given cognitive, psychiatric, and socio-demographic assessments that included both structured diagnostic interviews as well as self-reported questionnaires.

Substance use patterns (including CPD) were assessed with the Composite International Diagnostic Interview—Substance Abuse Module (CIDI-SAM), a structured, face-to-face diagnostic assessment designed to be administered by trained, lay interviewers^4^. This assessment procedure has been shown to be valid for adolescent subjects^5^.

All research protocols and consent forms were approved by institutional review boards of the University of Colorado. 712 unrelated, Caucasian-Non Hispanic subjects were genotyped from the CADD sample. Of this subsample, 348 reported smoking.

Genomic DNA was isolated from buccal cells using a modification of published procedures^6-8^. Taqman assays for allelic discrimination (Applied Biosystems, Foster City, CA) were used to determine SNP genotypes. QC performed on the genotyped sample (by sample and by SNP) excluded individuals with less than 50% genotypes (assumed poor quality DNA sample). All SNPs had greater than 95% genotype calling after exclusion of individuals with low quality DNA samples. All genotypes were called by two independent individuals.

References Cited:

1. Stallings MC, Corley RP, Hewitt JK, Krauter KS, Lessem JM, Mikulich SK, Rhee SH, Smolen A, Young SE, Crowley TJ (2003). A genome-wide search for quantitative trait loci influencing substance dependence vulnerability in adolescence. *Drug Alcohol Depend* 70, 295-307.
2. Stallings MC, Corley RP, Dennehey B, Hewitt JK, Krauter KS, Lessem JM, Mikulich-Gilbertson SK, Rhee SH, Smolen A, Young SE (2005). A genome-wide search for quantitative trait Loci that influence antisocial drug dependence in adolescence. *Arch Gen Psychiatry* 62, 1042-1051.
3. Defries, JC, Plomin, R, Fulker, DW (Eds.) (1994) Nature and nuture during middle childhood, vol 2. Blackwell Publishers, Oxford, UK.

4. Cottler LB, Keating SK (1990) Operationalization of alcohol and drug dependence criteria by means of a structured interview, *Recent Dev. Alcohol.* 8: 69–83.

5. Crowley TJ, Mikulich SK, Ehlers KM, Whitmore EA, MacDonald MJ (2001) Validity of structured clinical evaluations in adolescents with conduct and substance problems, *J. Am. Acad. Child Adolesc. Psychiatry* **40:** 265–273.

6. Freeman B, Powell J, Ball D, Hill L, Craig I, Plomin R (1997) DNA by mail: an inexpensive and noninvasive method for collecting DNA samples from widely dispersed populations, *Behav. Genet.* **27**: 251–2.

7. Lench N, Stanier P, Williamson R (1988) Simple non-invasive method to obtain DNA for gene analysis, *Lancet* **1:** 1356–1358.

8. Meulenbelt I, Droog S, Trommelen J, Boomsma I, Slagboom PE (1995) High-yield noninvasive human genomic DNA isolation method for genetic studies in geographically dispersed families and populations, *Am. J. Hum. Genet.* **57**: 1252–1254.

**7, 8. The American Cancer Society (ACS) Cancer Prevention Study-II Nutrition Cohort (CPS-II_CPD; CPS-II_LCA)**

The American Cancer Society (ACS) Cancer Prevention Study-II (CPS-II) Nutrition Cohort is a prospective study of cancer incidence and mortality among 86,404 men and 97,786 women. The Nutrition Cohort, which is described in detail elsewhere^1^, was initiated in 1992 as a subgroup of CPS-II, a prospective study of cancer mortality involving approximately 1.2 million Americans begun in 1982. Participants in the Nutrition Cohort were recruited from CPS-II members who resided in 21 states and were between the ages of 50 and 74 years. At enrollment in 1992/1993, participants completed a self-administered questionnaire that included demographic, medical, dietary, and lifestyle information. Follow-up questionnaires were sent to all living Nutrition Cohort members in 1997, and every two years after this to update exposure information and to ascertain newly diagnosed cancers. All aspects of the CPS-II Nutrition Cohort study are approved by the Emory University Institutional Review Board.

For the smoking population, all subjects were required to have smoked more than 100 cigarettes lifetime. A detailed description of this population appears elsewhere^2^. Cases were required to have smoked at least 30 cigarettes per day for at least five years. Controls smoked for at least one year during their lifetime and, in 1982 and 1992, reported having smoked fewer than 5 cigarettes per day, and in 1997, fewer than 10 cigarettes per day. Cases were selected to be balanced for gender whereas controls were predominantly female even after all available males were selected. For this meta-analysis, the CPS-II Nutrition cohort smoking cohort (CPS-II_CPD) contributed 2844 unrelated European-Americans (1458 heavy smokers and 1386 light smokers).

DNA was obtained from either a buffy coat or buccal cell sample collected from participants between 1998 and 2002. Genotyping was carried out using Illumina GoldenGate and Sequenom MassArray iPLEX technology. SNPs with a call rate of less than 95% and those for which Hardy-Weinberg equilibrium (HWE) was rejected (p<0.05) were excluded. DNA samples with call rate <90% were also excluded.

Participants who developed lung cancer between enrollment in 1992 and 2006 were identified either through self report on a follow-up questionnaire or through linkage with the National Death Index. The lung cancer diagnosis of the self reported cases was verified through medical records or linkage with state cancer registries. Controls were selected from a group of CPS-II participants for whom extensive genotyping had already been completed and who were cancer-free at the time of diagnosis of their matched case. Controls were matched to cases on age (± 2.5 years), gender, and sample type for DNA (buffy coat or buccal cell). All cases and controls were of European descent. For this meta-analysis, the CPS-II Nutrition cohort lung cancer population (CPS-II_LCA) contributed 699 unrelated cases and 748 unrelated controls.

Chronic pulmonary obstructive disease (COPD) status was also assessed among these participants, independent of lung cancer status. Participants were asked if they had ever been diagnosed with emphysema or chronic bronchitis by a physician in 1982, 1997, 1999, 2001, 2003 and 2005 and if they had ever been diagnosed with emphysema in 1992. Individuals who responded yes to these questions at any time were classified as cases whereas all others were defined as controls. For this meta-analysis, the CPS-II Nutrition cohort lung cancer population contributed 330 COPD cases and 1117 controls.

DNA was obtained from either a buffy coat or buccal cell sample collected from CPS-II Nutrition cohort participants between 1998 and 2002. Genotyping of the DNA samples was carried out using Illumina HumanHap550K, HumanHap610, or HumanHap 1 Million technologies. Genotypes with a call rate of less than 85%, more than 1 HapMap replicate error, more than a 3% (autosomal) or 5% (X chromosome) difference in call rate between genders, or more than 0.5% male AB frequency for the X chromosome, were excluded.

References Cited:

1. Calle EE, Rodriguez C, Jacobs EJ, et al. (2002) The American Cancer Society Cancer Prevention Study II Nutrition Cohort. *Cancer* 94:2490-2501.

2. Stevens VL, Bierut LJ, Talbot JT, et al. (2008) Nicotinic receptor gene variants influence susceptibility to heavy smoking. *Cancer Epidemiol Biomarkers Prev* 17:3517-3525.

**9. Evaluation of COPD Longitudinally to Identify Predictive Surrogate Endpoints (ECLIPSE)**

Subjects from the Evaluation of COPD Longitudinally to Identify Predictive Surrogate Endpoints (ECLIPSE) Study have been described previously^1^. Briefly, ECLIPSE is a multinational, 3-year, non-interventional investigation of COPD cases (GOLD Stages II – IV) and controls designed to identify factors predictive of progression of COPD in various COPD subtypes and to identify clinically relevant biomarkers for the prediction of disease progression. Cases were diagnosed by post-bronchodilator spirometry as GOLD Stage II (FEV1 < 80% predicted and FEV1FEV1/FVC < 0.7) or worse. Both cases and controls were self-reported white ethnicity, between ages 40-75 and were required to have at least a 10 pack-year smoking history. The number of cigarettes per day (CPD) was assessed based on the average for the overall smoking period as well as on the current CPD; the larger of the two values was used to define the CPD trait for analysis.

For this analysis, a total of 1719 Caucasian COPD cases and 172 non-COPD controls were used. For the cigarettes-per-day (CPD) phenotype analyzed here, ECLIPSE contributed 137 smokers with CPD < 10 cigarettes, 976 with 10 < CPD < 20, 431 with 20 < CPD < 30, and 347 with CPD > 30. The study obtained informed consent from participants and approval from the appropriate institutional review boards.

Genotyping was performed on the HumanHap 550 V3 at outside institutions. Two channel intensities from each cohort were brought into Beadstudio workspaces, and reclustering was performed using project samples. Subjects with a call rate of < 95% and SNPs with a call frequency < 95% were a priori removed. The remaining markers were then cleaned following Illumina guidelines (<http://www.illumina.com/downloads/GTDataAnalysis_TechNote.pdf>). Additional manual SNP reviews and reclustering were performed by the same four operators, with one operator (NB) reviewing all manual calls from all cohorts. Manual reviews were also performed for the cluster plots from the top association results reported in this manuscript. Individuals were excluded using the default settings for outlier removal^2^. The number of principal components to be included was determined using Tracy-Widom statistics with P-value < 0.05.

References Cited:

1. Vestbo J, Anderson W, Coxson HO, Crim C, Dawber F, Edwards L, Hagan G, Knobil K, Lomas DA, MacNee W, Silverman EK, Tal-Singer R, ECLIPSE investigators (2008). Evaluation of COPD Longitudinally to Identify Predictive Surrogate End-points (ECLIPSE). *Eur Respir J* 31:869-873.
2. Price AL, Patterson NJ, Plenge RM, et al. (2006) Principal components analysis corrects for stratification in genome-wide association studies. *Nat Genet* 38:904-9.

**10. GenMetS**

Subjects were drawn from a Health2000 study that includes 8028 subjects aged 30 or over and is a nationally representative sample of the adult Finnish population^1^. GenMetS is a subcohort of 2124 individuals selected for a case-control genome-wide association study on metabolic syndrome^2^; 918 cases were selected according the International Diabetes Federation Worldwide Definition of the Metabolic Syndrome and 1206 controls were selected for not carrying the trait. The subjects participated in a health interview conducted by Statistic Finland’s interview staff at the home of the participants. During the interview respondents were handed an information leaflet and an informed consent form that was returned after signing. The interview extensively examined factors influencing health, including a one-page questionnaire on smoking behavior. The number of cigarettes smoked daily (CPD) was queried by a question “How much do you daily smoke currently or did prior to quitting?” The respondent was asked to indicate the number of cigarettes smoked as an open-ended question. The genotyped sample included 1134 smokers who answered yes to the question “Have you smoked at least 100 times during your lifetime?”

DNA was extracted from a venous blood sample and genotyped by the Illumina 610 Quad V1 BeadChip at the Sanger Wellcome Trust Institute. This chip provides whole-genome SNP genotyping information with 598,203 SNP markers. The SNPs and samples were screened for the following: SNP clustering probability for each genotype > 95%, Call rate > 95% for both individuals and markers, MAF > 1%, and HWE p > 1*10^-6^. In addition, heterozygosity, gender checks and relatedness checks were performed and any discrepancies were removed. The SNPs included in the analyses, rs8034191, rs578776, rs621849, and rs8192475, all had genotyping success of nearly 100% (0-3 missing genotypes per SNP) and Hardy-Weinberg equilibrium test p-values of 0.41, 0.11, 0.43, and 0.63, respectively. The minor (MAF) and major alleles were G (34%) and A; A (32%) and G; G (37%) and A; and A (2.4%) and G, respectively.

References Cited:

1. Aromaa A, Koskinen,S. (eds) (2004) Health and functional capacity in Finland. Publications of the National Public Health Institute, KTL B12: Helsinki, Finland. <http://www.terveys2000.fi/julkaisut/baseline.pdf>
2. Keskitalo K, Broms U, Heliövaara M, Ripatti S, Surakka I, Perola M, Pitkäniemi J, Peltonen L, Aromaa A, Kaprio J. (2009) Association of serum cotinine level with a cluster of three nicotinic acetylcholine receptor genes (*CHRNA3*/*CHRNA5*/*CHRNB4*) on chromosome 15. *Human Molecular Genetics* 18:4007-12.

**11-13, 24-27. Health Professionals Follow-up Study (HPFS); Nurses’ Health Study (NHS)**

The seven analysis samples that contribute to this meta-analysis were derived from case-control cohort studies nested within two multi-site U.S. cohort studies, the Nurses’ Health Study (NHS) and the Health Professionals Follow-Up Study (HPFS). The NHS cohort was initiated in 1976 across 11 states with 121,700 female registered nurses aged 30-55. The HPFS cohort was initiated in 1986 across 50 states with 51,529 men in health professions aged 40-75. In both cohorts information on smoking behaviors, including ever-smoking and cigarettes-per-day, was collected at baseline and through subsequent biennial mail-in questionnaires. In addition, blood samples were collected between 1989 and 1990 in the NHS, and between 1993 and 1994 in the HPFS.

Nested case-control cohort studies were conducted for Type 2 diabetes (T2D), cardiovascular disease (CHD), and kidney stones (KS) in each of the two cohorts, and an additional case-control study of breast cancer (BrCa) was conducted in the NHS cohort. In the T2D study, controls were defined to be those free of diabetes at the time of diagnosis of the case, and were initially matched on year of birth, month of blood collection, and fasting status, with matched-pairs subsequently broken because not all subjects gave informed consent for the posting of their data on dbGaP^1^. In the CHD study, controls were randomly selected from participants who provided blood samples and did not experience CHD, with two controls for every case. Controls were matched on age, smoking, and month of blood draw. In the KS study, participants with a history of kidney stones and randomly selected controls were identified in two cycles from those with no history of cancer (cycles 1 and 2) or cardiovascular disease (cycle 1) who met age eligibility requirements (cycle 1: HPFS<71; NHS<66; cycle 2: <76)^2^ . Finally, in the BrCa study, cases and controls were limited to post-menopausal women, who were not diagnosed with breast cancer during follow up. Controls were postmenopausal women matched with cases by age and post-menopausal hormone use at blood draw^3^. All studies from which the samples are derived obtained informed consent from participants and approval from the appropriate institutional review boards.

Current and former smokers were selected for analysis of the cigarettes-per day trait. The NHS samples contributed 1646 (NHS-T2D), 748 (NHS-CHD), 254 (NHS-KS) and 1210 (NHS-BrCa) smokers. Cigarettes-per-day was measured using information from the baseline questionnaire and follow-up questionnaires through 2002, or the latest follow-up questionnaire available. The cigarettes-per-day trait reflects the average number of cigarettes per day over the period of observation, or pack-years/smoking duration. In the NHS samples, pack-years was calculated based on write-in values for cigarettes-per-day through 1982 and a categorical reporting of cigarettes-per-day after 1982^4^.

The HPFS samples contributed 1237 (HPFS-T2D), 699 (HPFS-CHD), and 218 (HPFS-KS) smokers. Cigarettes-per-day was measured using information from the baseline questionnaire in 1989, and follow-up questionnaires through 2002 or the latest follow-up questionnaire available. The cigarettes-per-day trait phenotype used in analysis was assigned based on a combination of the average number of cigarettes per day over the period of observation, or pack-years/smoking duration, and a self-reported maximum average number of cigarettes per day, reported categorically.

In all samples, DNA was derived from white blood cells. Analysis samples were restricted to subjects of European ancestry, and genotyping used the Affymetrix 6.0 (T2D and CHD studies), the Illumina 610Q (KS studies), and the Illumina 550 (BrCa study) platforms. Although exact protocols varied by sample, at a minimum DNA samples that did not meet a 90% completion threshold, and SNPs with low call rates (<90%), were dropped. Analyses based on principal components were conducted to assess self-reported race, and any self-reported "white" samples that had substantial similarity to non-European reference samples (either the HapMap YRI or CHB+JPT samples) were excluded.

References Cited:

1. Qi L, Cornelis MC, Kraft P, Stanya KJ, Kao WH, Pankow JS, Dupuis J, Florez JC, Fox CS, Paré G, Sun Q, Girman CJ, Laurie CC, Mirel DB, Manolio TA, Chasman DI, Boerwinkle E, Ridker PM, Hunter DJ, Meigs JB, Lee CH; Meta-Analysis of Glucose and Insulin-related traits Consortium (MAGIC); Diabetes Genetics Replication and Meta-analysis (DIAGRAM) Consortium, van Dam RM, Hu FB. (2010) Genetic variants at 2q24 are associated with susceptibility to type 2 diabetes. *Human Molecular Genetics*, in press.

2. Curhan GC, Taylor EN (2008). 24-h uric acid excretion and the risk of kidney stones. *Kidney International* 73: 489–496.

3. Hunter DH, Kraft P, Jacobs KB, Cox DG, Yeager M, Hankinson SE, Wacholder S, Wang Z, Welch R, Hutchinson A, Wang J, Yu K, Chatterjee N, Orr N, Willett WC, Colditz GA, Ziegler RG, Berg CD, Buys SS, McCarty CA, Feigelson HS, Calle EE, Thun MJ, Hayes RB, Tucker M, Gerhard DS, Fraumeni JF Jr, Hoover RN, Thomas G, Chanock SJ. (2007) A genome-wide association study identifies alleles in *FGFR2* associated with risk of sporadic postmenopausal breast cancer. *Nature Genetics* 39: 870-874.

4. Caporaso N, Gu F, Chatterjee N, Sheng-Chih J, Yu K, Yeager M, Chen C, Jacobs K, Wheeler W, Landi MT, Ziegler RG, Hunter DJ, Chanock S, Hankinson S, Bergen AW, Kraft P (2009). Genome-wide and candidate gene association study of cigarette smoking behaviors. *PLoS ONE* 4(2): e4653.

**14. Lung Health Study (LHS)**

The Utah Genetics of Addiction Project contributed two cohorts (LHS and Utah) from a study of genetic risk markers for nicotine dependence and chronic obstructive pulmonary disease (COPD)^1^. The Lung Health Study (LHS) cohort was drawn from a multi-site longitudinal study of COPD sponsored by the Division of Lung Disease of the National Heart, Lung and Blood Institute^2^. All LHS participants had COPD as determined by pulmonary function testing, and all were smoking at the time of recruitment. All participants were of European descent, and all had smoked more than 100 cigarettes lifetime. Cigarettes per day (CPD) was based on period of heaviest smoking lifetime. Study procedures were approved by the University of Utah IRB.

DNA was isolated from peripheral blood lymphocytes collected by the LHS Study Investigators supported by the NHLBI^2^. SNP genotyping methods were previously described^1^ and used either the SNPlex assay (Applied Biosystems) or TaqMan assay (Applied Biosystems). The call rates were 100% in the LHS cohort. SNPs genotyped by both the TaqMan and SNPlex methods in 236 individuals had a concordance rate > 99.7%.

References Cited:

1. Weiss RB, Baker TB, Cannon DS, von Niederhausern A, Dunn DM, Matsunami N, et al. (2008). A candidate gene approach identifies the CHRNA5-A3-B4 region as a risk factor for age-dependent nicotine addiction. *PLoS Genet,* 4(7), e1000125.

2. Anthonisen NR, Connett JE, Kiley JP, Altose MD, Bailey WC, Buist AS, et al. (1994). Effects of smoking intervention and the use of an inhaled anticholinergic bronchodilator on the rate of decline of FEV1. The Lung Health Study*. JAM*A, 272(19), 1497-1505.

**15. MD Anderson**

Study subjects (all self-reported Caucasians) from the U.T. M.D. Anderson Cancer Center (MDACC) are derived from a lung cancer case-control study that has been ongoing since 1991^1^. Cases were newly diagnosed, histologically-confirmed patients presenting at M.D. Anderson Cancer Center with the diagnosis of non-small cell lung cancer and who had not previously received treatment other than surgery. Controls were healthy individuals seen for routine care at Kelsey-Seybold Clinics; the largest physician group-practice plan in the Houston Metropolitan area^2^. Controls were frequency matched to cases according to their smoking behavior, age in 5 year categories, ethnicity, and sex. Former smoking controls were further frequency matched to former smoking cases according to the number of years since smoking cessation (in 5 year categories). The study protocols were approved by the Institutional Review Board of the U.T. M.D. Anderson Cancer Center. Informed consent was obtained from all patients. Epidemiologic data including smoking status were collected during an in-person interview using a structured questionnaire.

Genomic DNA was extracted from peripheral blood samples using the Human Whole Blood Genomic DNA Extraction Kit (Qiagen, Valencia, CA). Genotypes were generated by the Center for Inherited Disease Research for 317,498 polymorphic tagging SNPs using Illumina HumanHap300 v1.1 BeadChips and the Illumina Infinium II assay^3^. For this meta-analysis, MDACC contributed a sample of 2291 unrelated European-Americans (including 1136 cases and 250 controls). All subjects are ever smokers (smoked more than 100 cigarettes lifetime).

References Cited:

1. Spitz MR, Wei Q, Dong Q, Amos CI, Wu X (2003) Genetic susceptibility to lung cancer: the role of DNA damage and repair. *Cancer Epidemiol. Biomarkers Prev*. 12, 689-698.
2. Hudmon KS, et al. (1997) Identifying and recruiting healthy control subjects from a managed care organization: a methodology for molecular epidemiological case-control studies of cancer. *Cancer Epidemiol. Biomarkers Prev*. 6, 565-571.
3. Amos CI, Wu X, Broderick P, et al. (2008) Genome-wide association scan of tag SNPs identifies a susceptibility locus for lung cancer at 15q25.1. *Nat Genet.* 40:616–622.

**16-19. Munich Germany (MUC12SCS; MUC12SCTL; MUCMDCS; MUCMDCTL)**

Healthy unrelated volunteers of German descent (i.e., both parents German) were randomly selected from the general population of Munich, Germany, and contacted by mail. To exclude subjects with central neurological diseases and psychotic disorders or subjects who had first-degree relatives with psychotic disorders, several screenings were conducted before the volunteers were enrolled in the study. First, subjects who responded were screened by phone for the absence of neuropsychiatric disorders. Second, detailed medical and psychiatric histories were assessed for subjects and their first-degree relatives by using a semi-structured interview. Third, if no exclusion criteria were fulfilled, they were invited to a comprehensive interview including the Structured Clinical Interview for DSM-IV (SCID I and SCID II)^1,2^ to validate the absence of any lifetime psychotic disorder. Additionally, the Family History Assessment Module^3^ was conducted to exclude psychotic disorders among first-degree relatives. Furthermore, a neurological examination was conducted to exclude subjects with current CNS impairment. In the case that the volunteers were older than 60 years, the Mini Mental Status Test^4^ was performed to exclude subjects with possible cognitive impairment.

Individuals with schizophrenia were ascertained from the Munich area in Germany. Of this sample, 66% were of German descent and 34% were Caucasian middle Europeans. No evidence for ethnic stratification was observed after testing with the software STRUCTURE)^5^. Case participants had a DSM-IV and ICD-10 diagnosis of schizophrenia with the following subtypes: paranoid 77.2%, disorganized 16.3%, catatonic 2.8% and undifferentiated 3.7%. Detailed medical and psychiatric histories were collected, including a clinical interview using the SCID, to evaluate lifetime Axis I and II diagnoses. Four physicians and one psychologist rated the SCID interviews, and all measurements were double-rated by a senior researcher. Exclusion criteria included a history of head injury or neurological diseases. All case participants were outpatients or stable inpatients. Further details can be found in previous reports^6^.

Smoking behavior was grouped into current, former and never smokers. The number of cigarettes per day (CPD) as well as FTND was assessed for the period of heaviest smoking and for average use. For this meta-analysis, the German sample contributed unrelated European Caucasians (1052 healthy controls and 641 schizophrenia patients for MUCMD, and 235 controls and 421 schizophrenia patients for MUC12S).  All subjects were smokers and reported smoking 100 cigarettes lifetime. The study obtained informed consent from participants and approval from the appropriate institutional review boards.

DNA was obtained from peripheral blood. DNA concentration was adjusted using the PicoGreen quantitation reagent (Invitrogen, Karlsruhe, Germany), and 1 ng was genotyped using the iPLEX assay on the MassARRAY MALDI-TOF mass spectrometer (SEQUENOM, Hamburg, Germany). Genotyping call rates in cases and controls were all >97%. Allele frequencies were similar to CEU sample frequencies. A subsample of SNPs and DNA was genotyped twice to check for genotyping errors.

For this meta-analysis, MUC12S and MUCMD were each separated into schizophrenic cases (CS) and controls (CTL) prior to running association analyses, because schizophrenic patients are known to have different, heavy patterns of smoking compared to normal controls.

References Cited:

1. First MB, Spitzer RL, Gibbon M, Williams BW, Benjamin L (1990) Structured Clinical Interview for DSM-IV Axis II Personality Disorders (SCID-II). New York: Biometrics Research Department, New York State Psychiatric Institute.
2. First MB, Spitzer RL, Gibbon M, Williams JB (1995) Structured Clinical Interview for DSM-IV Axis I Disorders - Patient Edition (SCID - I/P, Version 2.0). New York: Biometrics Research Department, New York State Psychiatric Institute.
3. Rice JP, Reich T, Bucholz KK, Neuman RJ, Fishman R, Rochberg N, Hesselbrock VM, Nurnberger JI, Jr., Schuckit MA, Begleiter H (1995) Comparison of direct interview and family history diagnoses of alcohol dependence. *Alcohol Clin Exp Res* 19:1018-23.

4. Folstein MF, Folstein SE, McHugh PR. (1990) Mini-Mental-Status-Test. German Version: Kessler J, Folstein SE, Denzler P. Weinheim: Beltz.

5. Pritchard JK, Rosenberg NA (1999) Use of unlinked genetic markers to detect population stratification in association studies. *Am J Hum Genet* 65(1):220-8.

6. Van den Oord EJ, Rujescu D, Robles JR, Giegling I, Birrell C et al. (2006) Factor structure and external validity of the PANSS revisited. *Schizophr Res* 82: 213-23.

**20, 21. Nicotine Addiction Genetics Project and Australian Big Sibship Projects (NAG-Aus/BigSib; NAG-Finland)**

The study participants for the Nicotine Addiction Genetics Project (NAG) were enrolled at two different sites: the Queensland Institute of Medical Research (QIMR) in Australia and the University of Helsinki (UH) in Finland. Families for both the Australian and Finnish arms of the NAG were identified through smoking index cases by use of previously administered interview and/or questionnaire surveys of the community-based Australian and population-based Finnish registers of twins^1,2^. The Finnish arm of the NAG project (NAG-Fin) recruited families from the Finnish Twin Cohort, which consists of all Finnish twin pairs born between 1938 and 1957^3^. Families chosen for the Australian arm of the NAG study (NAG-Aus) were identified from two cohorts of the Australian Twin Panel, which included spouses of the older of these two cohorts. The ancestry of the Australian samples is predominantly Anglo-Celtic or northern European (>90%). We also used data obtained from a third Australian Community-based family study, the Australian Big Sibship (BigSib). The BigSib sample comprises families ascertained through the Australian Twin Panel selected for five or more offspring sharing both biological parents. Families for the BigSib sample were recruited from the same Australian Twin Panel sources as were the NAG Australian families, and phenotypic information was obtained using the same assessment protocol as for the NAG. Clinical data for both Australian and Finnish subjects were collected using a computer-assisted telephone diagnostic interview (CATI), and adaptation of the Semi-Structured Assessment for the Genetics of Alcoholism (SSAGA)^4,5^ for telephone administration. The tobacco section of the CATI was derived from the Composite International Diagnostic Interview (CIDI)^6^ and incorporated standard FTND, DSM-III-R, and DSM-IV assessments of nicotine dependence. It also included a detailed history of cigarette and other tobacco use, including quantity and frequency of use for current, most recent, and heaviest period of use. The measure examined for the purposes of this study was the number of cigarettes smoked per day, during heaviest period of use. All data-collection procedures were approved by institutional review boards at Washington University (WU), the QIMR, and the Ethics committee of the Hospital District of Helsinki and Uusimaa, including the use of appropriate and approved informed-consent procedures.

For this meta-analysis, NAG/BigSib-Aus combined sample contributed information from a total of 1329 unrelated adult subjects (about 40% women; including 45% from the BigSib sample), 18-82 years of age (mean age: 44 years) at the time of assessment; including 592 who reported smoking 10 or fewer cigarettes, 489 subject who reported smoking 20 to 39, and 248 Australians who reported smoking 40 or more cigarettes during their heaviest period of smoking. Participants gave informed consent for an interview, for providing a blood sample for DNA extraction and cell lines, and for the sharing of their anonymous clinical and genotypic records with scientists outside of the NAG and/or BigSib research teams of investigators.

Analyzed as a separate sample, NAG-Fin contributed information from a total of 207 unrelated adult subjects (about 40% women); 39-93 years of age (Mean age: 58 years) at the time of assessment; including 29 who reported smoking 10 or fewer cigarettes, 133 who reported smoking more than 10 but less than 26, 32 who reported smoking 26 to 39 cigarettes, and 13 Finns who reported smoking 40 or more cigarettes during their period of heaviest smoking. All subjects had at least experimented with cigarettes in the past. Participants gave informed consent for an interview, for providing a blood sample for DNA extraction, and for the sharing of their anonymous clinical and genotypic records with scientists outside of the NAG research teams of investigators.

References Cited:

1. Saccone SF, Pergadia ML, Loukola A, Broms U, Montgomery GW, Wang JC, Agrawal A, Dick DM, Heath AC, Todorov AA, Maunu H, Heikkila K, Morley KI, Rice JP, Todd RD, Kaprio J, Peltonen L, Martin NG, Goate AM, Madden PAF (2007) Genetic linkage to chromosome 22q12 for a heavy-smoking quantitative trait in two independent samples. *Am J Hum Genet* 80:856-866.
2. Loukola A, Broms U, Maunu H, Widén E, Heikkilä K, Siivola M, Salo A, Pergadia ML, Nyman E, Sammalisto S, Perola M, Agrawal A, Heath AC, Martin NG, Madden PAF, Peltonen L, Kaprio J (2008) Linkage of nicotine dependence and smoking behavior on 10q, 7q and 11p in twins with homogenous genetic background. *The Pharmacogenomics Journal* 8:209-219.
3. Kaprio J, Koskenvuo M. Genetic and environmental factors in complex diseases: the older Finnish Twin Cohort (2002) *Twin Res* 5:358-365.
4. Bucholz KK, Cadoret R, Cloninger CR, Dinwiddie SH, Hesselbrock VM, Nurnberger JI Jr, Reich T, Schmidt I, Schuckit MA (1994) A new, semi-structured psychiatric interview for use in genetic linkage studies: a report on the reliability of the SSAGA. *J Stud Alcohol* 55:149-158.
5. Hesselbrock M, Easton C, Bucholz KK, Schuckit M, Hesselbrock V (1999) A validity study of the SSAGA—a comparison with the SCAN. *Addiction* 94:1361-1370.
6. Cottler LB, Robins LN, Grant BF, Blaine J, Towle LH, Witthen HU, Sartorius N (1991) The CIDI-core substance abuse and dependence questions: cross-cultural and nosological issues: the WHO/ADAMHA field trial. *Br J Psychiatry* 159:653-658.

**22, 23. National Cancer Institute (NCI-EAGLE; NCI-PLCO)**

We have conducted a genome-wide association study (GWAS) of common genetic variants to identify genetic markers of susceptibility to lung cancer and smoking. The initial scan was conducted in ~ 2,000 lung cancer cases and 2,000 controls from the Environment and Genetics in Lung Cancer Etiology (EAGLE) Study, a population-based, case-control study, as well as ~850 lung cancer cases and ~850 controls from the Prostate, Lung, Colon and Ovary Screening Trial (PLCO), which is a cohort design. This group was scanned with a set of 500,000 SNPs with minor allele frequencies (MAF > 5%) that serve as markers for approximately 90% of all common SNPs in Caucasians using the Illumina 550 duo platform.

EAGLE was conducted in the Lombardy region of Italy between 2002 and 2005, including 2101 verified incident primary lung cancers and 2120 healthy, population-based controls. Participants were ages 35 to 79 years at diagnosis (cases) or enrollment for interview (controls). Epidemiological data and DNA specimens were collected from 98.4% and 97.3% of the cases, respectively. Extensive epidemiological data were collected through both a Computer Assisted Personal interview (CAPI) and a self-administered questionnaire. A total of 3,111 smokers (1447 current and 1664 former smokers) from EAGLE participated in the initial scan.

Detailed data on tobacco smoking included: information on number of cigarettes and other tobacco products per day, averaged over each smoking period of life and during the last year, age at first cigarette, at initiation (i.e., at least once per week) and quitting; the number of quitting attempts and time between attempts, inhalation habits, passive smoking during childhood, at home and in the workplace, and self-reported willingness to quit smoking. Smoking status (classification into never, ever, and current smoking status) was established by review of smoking data. Ever smokers all had ‘smoked greater than 100 cigarettes during their lifetime with a frequency of one or more cigarettes per week, establishing their status as smokers. Former smokers had indicated that in addition ‘during the last 6 months’ they had not been smoking, i.e. ‘not at all or less than one cigarette per week’. Current smokers, in addition to their status as smokers, indicated that during the last six months they smoked at least one cigarette per week. This smoking information was cross validated through checks for concordance with the other smoking information listed above. A variety of behavioral rating scales related to smoking were collected, including the Fagerström Test for Nicotine Dependence (FTND), nicotine withdrawal, Beck Depression Inventory and others^1^.

Study participants from PLCO include 1442 subjects from the lung cancer study (750 current smokers and 692 former smokers) and 1376 subjects from the prostate cancer study (213 current smokers and 1163 former smokers). In the replication phase, there were 516 subjects who were all bladder cancer cases comprised of 99 current smokers and 417 former smokers. Smoking behaviors were measured by baseline questionnaire (BQ) in the PLCO (administered from 1994-2001)^2^. Former smokers were defined as ever-smokers who did not smoke regularly at BQ and were asked to report the age at which they stopped smoking regularly. Ever smokers provided information on the number of cigarettes they smoked per day, in categories (1-10, 11-20, 21-30, 31-40, 41-60, 61-80, over 80). For continuous analyses we assigned subjects to the midpoint of their category (or 90 cigarettes per day for over 80).

DNA was obtained from germline material derived from peripheral blood. A portion of the PLCO controls derived DNA from buccal DNA (also, buccal was the source for a very small group of EAGLE subjects who lacked a blood sample). The EAGLE sample consisted of 1,955 cases and 2,019 controls (N=3,974), whereas the PLCO had 816 cases and 859 controls (N=1,675). Institutional Review Board approval was obtained for all studies, and informed consent was obtained from all participants.

PLCO and EAGLE samples were genotyped at the Center for Inherited Disease Research (CIDR) as part of the Gene Environment Association Studies initiative (GENEVA) funded by the National Human Genome Research Institute. Data were released for 5,620 of 5,727 (98%) attempted study samples. Study samples, including 32 blind duplicates, were plated and genotyped together with 124 HapMap controls (66 CEU; 58 YRI). Genotyping was performed using Illumina HumanHap550v3_B BeadChips (Illumina, San Diego, CA, USA) and the Illumina Infinium II assay protocol^3^. Allele cluster definitions for each SNP were determined using Illumina BeadStudio Genotyping Module version 3.1.14 and the combined intensity data from 95% of the samples. The resulting cluster definitions were used on all samples. Genotypes were not called if the quality threshold (Gencall score) was below 0.15. Genotypes were released by CIDR for 560,505 (99.83% of attempted) SNPs. Genotypes were not released for SNPs that were not called by BeadStudio, had call rates less than 85%, more than 1 HapMap replicate error, more than a 3% (autosomal) or 5% (X chromosome) difference in call rate between genders, or more than 0.5% male AB frequency for the X chromosome. Y and XY SNPs were manually reviewed and clusters adjusted or genotypes dropped as appropriate. Intensity data were released for all attempted SNPs. The mean non-Y SNP call rate and mean sample call rate was 99.8% for the released CIDR dataset. Blind duplicate reproducibility was 99.993%. The dataset is available on dbGaP (http://www.ncbi.nlm.nih.gov/sites/entrez?Db=gap, Study Accession: phs000093.v1.pl).

References Cited:

1. Hayes RB, et al. (2000) Etiologic and early marker studies in the prostate, lung, colorectal and ovarian (PLCO) cancer screening trial. *Control Clin Trials* 21, 349S-355S.

2. Prorok PC, et al. (2000) Design of the Prostate, Lung, Colorectal and Ovarian (PLCO) Cancer Screening Trial. *Control Clin Trials* 21, 273S-309S .

3. Gunderson KL, Steemers FJ, Ren H, Ng P, Zhou L, Tsan C, Chang W, Bullis D, Musmacker J, King C, Lebruska LL, Barker D, Oliphant A, Kuhn KM, Shen R. (2006) Whole-genome genotyping. *Methods Enzymol.* 2006;410:359-76. Review.

**11-13, 24-27. Health Professionals Follow-up Study (HPFS); Nurses’ Health Study (NHS)**

The seven analysis samples that contribute to this meta-analysis were derived from case-control cohort studies nested within two multi-site U.S. cohort studies, the Nurses’ Health Study (NHS) and the Health Professionals Follow-Up Study (HPFS). The NHS cohort was initiated in 1976 across 11 states with 121,700 female registered nurses aged 30-55. The HPFS cohort was initiated in 1986 across 50 states with 51,529 men in health professions aged 40-75. In both cohorts information on smoking behaviors, including ever-smoking and cigarettes-per-day, was collected at baseline and through subsequent biennial mail-in questionnaires. In addition, blood samples were collected between 1989 and 1990 in the NHS, and between 1993 and 1994 in the HPFS.

Nested case-control cohort studies were conducted for Type 2 diabetes (T2D), cardiovascular disease (CHD), and kidney stones (KS) in each of the two cohorts, and an additional case-control study of breast cancer (BrCa) was conducted in the NHS cohort. In the T2D study, controls were defined to be those free of diabetes at the time of diagnosis of the case, and were initially matched on year of birth, month of blood collection, and fasting status, with matched-pairs subsequently broken because not all subjects gave informed consent for the posting of their data on dbGaP^1^. In the CHD study, controls were randomly selected from participants who provided blood samples and did not experience CHD, with two controls for every case. Controls were matched on age, smoking, and month of blood draw. In the KS study, participants with a history of kidney stones and randomly selected controls were identified in two cycles from those with no history of cancer (cycles 1 and 2) or cardiovascular disease (cycle 1) who met age eligibility requirements (cycle 1: HPFS<71; NHS<66; cycle 2: <76)^2^ . Finally, in the BrCa study, cases and controls were limited to post-menopausal women, who were not diagnosed with breast cancer during follow up. Controls were postmenopausal women matched with cases by age and post-menopausal hormone use at blood draw^3^. All studies from which the samples are derived obtained informed consent from participants and approval from the appropriate institutional review boards.

Smokers, current and former, were selected for analysis of the cigarettes-per day trait. The NHS samples contributed 1646 (NHS-T2D), 748 (NHS-CHD), 254 (NHS-KS) and 1210 (NHS-BrCa) smokers. Cigarettes-per-day was measured using information from the baseline questionnaire and follow-up questionnaires through 2002, or the latest follow-up questionnaire available. The cigarettes-per-day trait reflects the average number of cigarettes per day over the period of observation, or pack-years/smoking duration. In the NHS samples, pack-years was calculated based on write-in values for cigarettes-per-day through 1982 and a categorical reporting of cigarettes-per-day after 1982^4^.

The HPFS samples contributed 1237 (HPFS-T2D), 699 (HPFS-CHD), and 218 (HPFS-KS) smokers. Cigarettes-per-day was measured using information from the baseline questionnaire in 1989, and follow-up questionnaires through 2002 or the latest follow-up questionnaire available. The cigarettes-per-day trait phenotype used in analysis was assigned based on a combination of the average number of cigarettes per day over the period of observation, or pack-years/smoking duration, and a self-reported maximum average number of cigarettes per day, reported categorically.

In all samples, DNA was derived from white blood cells. Analysis samples were restricted to subjects of European ancestry, and genotyping used the Affymetrix 6.0 (T2D and CHD studies), the Illumina 610Q (KS studies), and the Illumina 550 (BrCa study) platforms. Although exact protocols varied by sample, at a minimum DNA samples that did not meet a 90% completion threshold, and SNPs with low call rates (<90%), were dropped. Analyses based on principal components were conducted to assess self-reported race and any self-reported "white" samples that had substantial similarity to non-European reference samples (either the HapMap YRI or CHB+JPT samples) were excluded.

References Cited:

1. Qi L, Cornelis MC, Kraft P, Stanya KJ, Kao WH, Pankow JS, Dupuis J, Florez JC, Fox CS, Paré G, Sun Q, Girman CJ, Laurie CC, Mirel DB, Manolio TA, Chasman DI, Boerwinkle E, Ridker PM, Hunter DJ, Meigs JB, Lee CH; Meta-Analysis of Glucose and Insulin-related traits Consortium (MAGIC); Diabetes Genetics Replication and Meta-analysis (DIAGRAM) Consortium, van Dam RM, Hu FB. (2010) Genetic variants at 2q24 are associated with susceptibility to type 2 diabetes. *Human Molecular Genetics*, in press.

2. Curhan GC, Taylor EN (2008). 24-h uric acid excretion and the risk of kidney stones. *Kidney International* 73: 489–496.

3. Hunter DH, Kraft P, Jacobs KB, Cox DG, Yeager M, Hankinson SE, Wacholder S, Wang Z, Welch R, Hutchinson A, Wang J, Yu K, Chatterjee N, Orr N, Willett WC, Colditz GA, Ziegler RG, Berg CD, Buys SS, McCarty CA, Feigelson HS, Calle EE, Thun MJ, Hayes RB, Tucker M, Gerhard DS, Fraumeni JF Jr, Hoover RN, Thomas G, Chanock SJ. (2007) A genome-wide association study identifies alleles in *FGFR2* associated with risk of sporadic postmenopausal breast cancer. *Nature Genetics* 39: 870-874.

4. Caporaso N, Gu F, Chatterjee N, Sheng-Chih J, Yu K, Yeager M, Chen C, Jacobs K, Wheeler W, Landi MT, Ziegler RG, Hunter DJ, Chanock S, Hankinson S, Bergen AW, Kraft P (2009). Genome-wide and candidate gene association study of cigarette smoking behaviors. *PLoS ONE* 4(2): e4653.

**28. National Youth Survey – Family Study (NYSFS; originally “National Youth Survey”)**

The National Youth Survey began in 1976. At that time 1,725 adolescents between the ages of 11 and 17 years old as well as one of their parents were interviewed. Participants were chosen by a scientific method designed to select individuals who were representative of the national population. It was a sample of households with all children between 11 and 17 within a chosen household recruited. It is a longitudinal study, with 12 waves of interviews conducted so far. DNA was collected as part of wave 10 interviews^1-3^. CPD was defined as the maximum number reported across the public waves (2-7). Subjects ranged from youth to young adults at that time.

DNA was collected for 1071 individuals, 20 of whom have mostly missing phenotype information and were thus excluded, so genotypes and phenotypes were used for 1051 individuals. Total recruited was 1725, but there has been a low level of attrition thru time (there is no evidence for any systematic trends in either attrition or DNA collection refusal). After selection for unrelated individuals, 548 smokers were phenotyped/genotyped. All research protocols and consent forms were approved by institutional review boards of the University of Colorado.

DNA was derived from buccal cells. Taqman assays for allelic discrimination (Applied Biosystems, Foster City, CA) were used to determine SNP genotypes. QC performed on the genotyped sample (by sample and by SNP) excluded individuals with less than 50% genotypes (assumed poor quality DNA sample). All SNPs had greater than 95% genotype calling after exclusion of individuals with low quality DNA samples. All genotypes were called by two independent individuals.

References Cited:

1. **Elliott DS, Huizinga D, Ageton SS (1985) Explaining Delinquency and Drug Use. Beverly Hills, CA: Sage Publications.**
2. **Elliott DS, Huizinga D, Menard S (1989) Multiple Problem Youth: Delinquency, Drugs and Mental Health Problems. New York, NY: Springer.**
3. Hoft NR, Corley RP, Schlaepfer IR, McQueen MB, Huizinga D, Menard S, Ehringer MA. (2009) Genetic association of the CHRNA6 and CHRNB3 genes with tobacco dependence in a nationally representative sample*. Neuropsychopharmacology*. 34(3):698-706.

**29. UK Lung Cancer GWAS Phase II** **(UK_Phase_II)**

UK Lung Cancer GWAS Phase II is part of a case-control study aimed at identifying genetic variants associated with lung cancer^1^. 5,470 self reported British residents with European ancestry were available for the CGASP lung cancer case-control meta-analysis. 2,465 cases were ascertained through the Genetic Lung Cancer Predisposition Study (GELCAPS). Blood samples were obtained from 3,005 healthy individuals recruited to the National Cancer Research Network genetic epidemiological studies, the National Study of Colorectal Cancer (NSCCG; 1999-2006; N = 541), GELCAPS (1999-2004; N = 1,492); and the Royal Marsden Hospital Trust/Institute of Cancer Research Family History and DNA Registry (1999-2004; N = 972). These controls were the spouses or unrelated friends of patients with malignancies. None had a personal history of malignancy at time of ascertainment.

For the CGASP cigarette per day (CPD) phenotype meta-analysis, 3,134 smokers from UK Lung Cancer GWAS Phase II were included. The smokers are defined as those who have smoked ≥ 100 cigarettes lifetime. The number of CPD recorded was the average number of CPD during the period of smoking.

DNA was extracted from samples using conventional methodologies and quantified using PicoGreen (Invitrogen, Carlsbad, USA). Phase II genotyping was carried out using Illumina Infinium custom arrays consisting of 33,060 SNPs according to the manufacturer's protocols. The stepwise SNP selection procedure and the quality control procedures applied to the samples and SNPs are detailed in a prior paper^1^. Briefly, DNA samples with GenCall scores <0.25 at any locus were considered “no calls”. A DNA sample was deemed to have failed if it generated genotypes at <95% of loci. A SNP was deemed to have failed if fewer than 95% of DNA samples generated a genotype at the locus. To ensure quality of genotyping, a series of duplicate samples were genotyped and cases and controls were genotyped in the same batches.

References cited:

1. Broderick P, Wang Y, Vijayakrishnan J, Matakidou A, Spitz MR, Eisen T, Amos CI, Houlston RS. (2009) [Deciphering the impact of common genetic variation on lung cancer risk: a genome-wide association study.](http://www.ncbi.nlm.nih.gov/pubmed/19654303?itool=EntrezSystem2.PEntrez.Pubmed.Pubmed_ResultsPanel.Pubmed_RVDocSum&ordinalpos=2) *Cancer Res*. 69(16):6633-41.

**30.** **The Utah Genetics of Addiction Project (Utah)**

The Utah Genetics of Addiction Project contributed two cohorts (LHS and Utah) from a study of genetic risk markers for nicotine dependence and chronic obstructive pulmonary disease (COPD)^1^. The UT cohort was made up of respondents to community advertising for persons who had smoked more than 100 cigarettes lifetime plus a subset of the Lung Health Study (LHS) participants originally recruited in Utah; these Utah LHS participants were excluded from the LHS cohort. UT participants were not drawn from a psychiatric treatment population, and no medical or behavioral treatments were offered as part of the study. UT volunteers were not excluded simply because they had a lifetime diagnosis of psychosis or Bipolar Disorder, but they were excluded if their current mental status made it impossible for them to complete the questionnaires or interviews. Pulmonary function testing determined 62% of the UT cohort had COPD. Of UT participants, 43% had not smoked for at least 2 years prior to participation in the study. All UT participants were of European descent, and all had smoked more than 100 cigarettes lifetime. Study procedures were approved by the University of Utah IRB.

DNA was isolated from peripheral blood lymphocytes collected in Salt Lake City, UT (UT cohort). SNP genotyping methods were previously described^1^ and used either the SNPlex assay (Applied Biosystems) or TaqMan assay (Applied Biosystems). The call rate was 99.9%; and SNPs genotyped by both the TaqMan and SNPlex methods in 236 individuals had a concordance rate > 99.7%.

References Cited:

1. Weiss RB, Baker TB, Cannon DS, von Niederhausern A, Dunn DM, Matsunami N, et al. (2008). A candidate gene approach identifies the CHRNA5-A3-B4 region as a risk factor for age-dependent nicotine addiction*. PLoS Genet*, 4(7), e1000125.

**31. The Mid-South Tobacco Family Study (UVa-MSTF)**

The Mid-South Tobacco Family (MSTF) Study is a family-based study to identify susceptibility loci and genes for nicotine dependence (ND). Participants were recruited primarily from the states of Tennessee, Mississippi, and Arkansas during 1999–2004. Proband smokers were required to be at least 21 years of age, to have smoked for at least the last 5 years, and to have consumed an average of 20 cigarettes per day for the last 12 months. Once a proband was identified, additional siblings and biological parents were recruited whenever possible, regardless of smoking status. If biological parents were not available, we attempted to recruit at least three full sibs per family. Detailed information of recruitment and characteristics of study participants have been reported previously^1-3^. Together, a total of 200 European-American and 402 African-American families were recruited for the MSTF study. The study obtained informed consent from participants and approval from the appropriate institutional review boards.

A total of 263 biologically unrelated smokers from the 200 European-American families were selected for this meta-analysis study. If both parents were smokers in a family, we selected both parents into the sample. For those families with only one parent smoking or those with no parent smoking, we randomly selected one smoker per family without any restriction. More than 90% were “current” smokers at the recruitment and 67% were female smokers.

DNA was derived from cell lines maintained by the Rutgers University Cell and DNA Repository following stringent quality control and assurance procedures ([www.rucdr.org](http://www.rucdr.org/)). Genotyping of the DNA samples was carried out using TaqMan technique, and detailed procedures on both genotyping and data cleaning have been described previously^4^.

References Cited:

1. Li MD, Beuten J, Ma JZ, Payne TJ, Lou XY, Garcia V, Duenes AS, Crews KM, Elston RC (2005). Ethnic- and gender-specific association of the nicotinic acetylcholine receptor 4 subunit gene (CHRNA4) with nicotine dependence. *Human Molecular Genetics* 14(9):1211-9.
2. Li MD, Payne TJ, Ma JZ, Lou XY, Zhang D, Dupont RT, Crews KM, Somes G, Williams NJ, Elston RC (2006). A genomewide search finds major susceptibility loci for nicotine dependence on chromosome 10 in African Americans. *Am J Hum Genet.* 79(4):745-51.
3. Li MD, Ma JZ, Payne TJ, Lou XY, Zhang D, Dupont RT, Elston RC (2008). Genome-wide linkage scan for nicotine dependence in European Americans and its converging results with African Americans in the Mid-South Tobacco Family sample. *Molecular Psychiatry* 13(4):407-16.
4. Li MD, Xu Q, Lou XY, Payne TJ, Niu T, Ma JZ (2010). Association and interaction analysis of variants in CHRNA5/CHRNA3/CHRNB4 gene cluster with nicotine dependence in African and European Americans. *Am J Med Genet B Neuropsychiatr Genet.* 153B(3):745-56.

**32. Virginia Adult Twin Study of Psychiatric and Substance Use Disorder (VA-twin)**

The VA-Twins were selected from the Virginia Adult Twin Study of Psychiatric and Substance Use Disorder, which was a population-based epidemiology study. Tobacco smoking and nicotine dependence were assessed by the Fagerström Tolerance Questionnaire (FTQ) and/or Fagerström Test for Nicotine Dependence (FTND) during the time of heaviest lifetime nicotine use. In this study, only regular smokers (defined as those who used at some point in their lives an average of at least seven cigarettes per week for a minimum of four weeks) were included (N = 2388). One subject from each twin pair was selected, and all subjects were of Caucasian ancestry. The study obtained informed consent from participants and approval from the institutional review board of Virginia Commonwealth University. DNA was extracted from buccal brushes. Genotyping was performed with the TaqMan genotyping method. To ensure the quality of genotyping, negative control samples were included in each plate. Genotypes were scored using a semi-automated protocol.

References Cited:

1. Chen X, Chen J, Williamson VS, An SS, Hettema JM, Aggen SH, Neale MC, Kendler KS (2009). Variants in nicotinic acetylcholine receptors alpha5 and alpha3 increase risks to nicotine dependence. *Am J Med Genet B Neuropsychiatr Genet*. 150B(7):926-33.
2. Kendler KS, Myers J, Prescott CA (2007) Specificity of genetic and environmental risk factors for symptoms of cannabis, cocaine, alcohol, caffeine, and nicotine dependence. *Arch.Gen.Psychiatry* 64(11): 1313-1320.
3. van den Oord EJ, Jiang Y, Riley BP, Kendler KS, Chen X (2003) FP-TDI SNP scoring by manual and statistical procedures: a study of error rates and types. *Biotechniques* 34: 610-6, 618-20, 622 passim.

**33. Family Health Study and the Women’s Epidemiology of Lung Disease Study (WSU)**

Data are from two population-based, case-control studies of lung cancer detailed in a recent publication: the Family Health Study (FHS studies I, II and III) and the Women’s Epidemiology of Lung Disease (WELD) Study^1^. Only Caucasian subjects with DNA from sources other than tissue blocks were included in these analyses. All studies were conducted by the same study staff using identical procedures, with cases ascertained through the population-based Metropolitan Detroit Cancer Surveillance System, an NCI-funded SEER registry. Studies differed only in the eligibility of cases, with the FHS focused on never smokers and cases diagnosed before age 50 years and the WELD study focusing on women. Only non small cell lung cancer (NSCLC) histology cases were included in the WELD study. Population-based controls were chosen using random digit dialing methods. All study controls were frequency matched to cases by 5-year age group, sex and race. Institutional Review Board approval was obtained for all studies, and informed consent was obtained from all participants.

Individuals who had smoked at least 100 cigarettes in their lifetime were designated as smokers. These subjects were also asked for the average number of cigarettes per day they smoked and the total number of smoking years. All subjects were asked if they had ever been diagnosed by a physician as having emphysema, chronic obstructive pulmonary disease (COPD) or chronic bronchitis. Individuals reporting one or more of these conditions were considered to have COPD.

DNA was extracted from whole blood or buccal cells (buccal swab or mouthwash sample). DNA was isolated from blood using a Gentra AutoPure Kit (Qiagen, Valencia, CA), buccal swabs with the BuccalAmp DNA Extraction Kit (Epicentre Technologies, Madison, WI) and mouthwash samples with the Gentra Puregene Kit (Qiagen). TaqMan Genotyping Assays (Applied Biosystems, Foster City, CA) were used to detect polymorphisms. DNA isolated from buccal cells was pre-amplified in an outer PCR reaction for added sensitivity. Either 25 ng DNA or 1 µl of the outer nest was amplified, with primers designed using Primer Express software (Applied Biosystems), and detected using an AB 7900 Sequence Detection System (Applied Biosystems). For quality control, 5% of the products were sequenced and 10% were directly repeated.

References Cited:

1. Schwartz AG, Cote ML, Wenzlaff AS, Amos, CI. (2009) Racial differences in the association between SNPs on 15q25.1, smoking behavior, and risk of non-small cell lung cancer *J Thorac Oncol* 4(10):1195-201.

**34. Yale Genetics of Cocaine Dependence, Genetics of Opioid Dependence and Genetics of Alcohol Dependence (YALE-UConn)**

Subjects were recruited from substance abuse treatment centers and through advertisements at the University of Connecticut Health Center, Yale University School of Medicine, the Medical University of South Carolina, the University of Pennsylvania, and McLean Hospital (Harvard Medical School). The sample was recruited for substance abuse outcomes, including cocaine, opioid, alcohol, and nicotine dependence. Individuals were excluded if diagnosed with Axis I major psychotic illness (e.g., schizophrenia or schizoaffective disorder). The study protocol was approved by the institutional review board at each clinical site. After complete description of the study to the subjects, written informed content was obtained. Genetic studies of substance dependence disorders and related traits in a subset of this sample have been published ^1-3^.

Subjects were interviewed using an electronic version of the Semi-Structured Assessment for Drug Dependence and Alcoholism (SSADDA)^4,5^ to derive diagnoses for lifetime nicotine, cocaine, opioid, and alcohol dependence according to DSM-IV criteria. The CPD phenotype was determined by the question “When you were smoking regularly, how many cigarettes did you usually smoke in a day?” The sample of unrelated individuals contains 912 individuals of European ancestry with genotype and phenotype data. All are smokers.

DNA was primarily extracted from immortalized cell lines or blood samples, with a small number from saliva. SNP genotyping was performed at Yale University using a closed-tube fluorescent TaqMan 5'-nuclease allelic discrimination assay ordered as “assays-on-demand” (Applied Biosystems Inc., Foster City, CA). Fluorescence plate reads and genotype calls were made using ABI 7900 Sequence Detection Systems. Two nanograms of genomic DNA were PCR amplified in 384-well plates using a 2-μl reaction volume. The insertion/deletion marker was genotyped by PCR amplification followed by agarose gel size fractionation.

At least two blank wells and two duplicate samples (for the purposes of cross-run confirmation of genotype assignment) were included in each 96-well plate. At least 8% of genotypes were repeated for quality control. The results were compared for verification. Results for 28 individuals for which the genotyping completely failed on the 3 SNPs were removed from analysis.

References Cited:

1. Gelernter J, Panhuysen C, Weiss R, Brady K, Hesselbrock V, et al (2005) Genomewide linkage scan for cocaine dependence and related traits: Linkages for a cocaine-related trait and cocaine-induced paranoia. *Am J Med Genet Part B (Neuropsychiatric Genetics)* 136B:45–52.
2. Zhang H, Kranzler HR, Weiss RD, Luo X, Brady KT et al. (2009). [Pro-opiomelanocortin gene variation related to alcohol or drug dependence: evidence and replications across family- and population-based studies.](http://www.ncbi.nlm.nih.gov/pubmed/19217079?itool=EntrezSystem2.PEntrez.Pubmed.Pubmed_ResultsPanel.Pubmed_RVDocSum&ordinalpos=26) *Biol Psychiatry* 66:128-136.
3. Gelernter J, Yu Y, Weiss R, Brady K, Panhuysen C et al. (2006) [Haplotype spanning TTC12 and ANKK1, flanked by the DRD2 and NCAM1 loci, is strongly associated to nicotine dependence in two distinct American populations.](http://www.ncbi.nlm.nih.gov/pubmed/17085484?itool=EntrezSystem2.PEntrez.Pubmed.Pubmed_ResultsPanel.Pubmed_RVDocSum&ordinalpos=73) *Hum Mol Genet* 15:3498-3507.
4. Pierucci-Lagha A, Gelernter J, Chan G, Arias A, Cubells JF, et al. (2007). [Reliability of DSM-IV diagnostic criteria using the Semi-structured Assessment for Drug Dependence and Alcoholism (SSADDA).](http://www.ncbi.nlm.nih.gov/pubmed/17590536?itool=EntrezSystem2.PEntrez.Pubmed.Pubmed_ResultsPanel.Pubmed_RVDocSum&ordinalpos=60) *Drug Alcohol Depend* 91:85-90.
5. Pierucci-Lagha A, Gelernter J, Feinn R, Cubells JF, Pearson D, et al. (2005) [Diagnostic reliability of the Semi-structured Assessment for Drug Dependence and Alcoholism (SSADDA).](http://www.ncbi.nlm.nih.gov/pubmed/15896927?itool=EntrezSystem2.PEntrez.Pubmed.Pubmed_ResultsPanel.Pubmed_RVDocSum&ordinalpos=102) *Drug Alcohol Depend* 80:303-312.

**Notes on Consortium Overlap**

The following datasets were also used in the recently published papers on smoking meta-analyses from the European Network of Genetic and Genomic Epidemiology (ENGAGE) consortium^1^ and The Tobacco and Genetics (TAG) consortium^2^.

GenMetS was used in ENGAGE^1^ (N = 648 smokers and 396 heavy/light smokers are used in the present study). NHS-BrCa was used in TAG^2^ (N = 1210 smokers and 664 heavy/light smokers are used in the present study). Munich was used in the replication phase for ENGAGE^1^ (N= 2349 smokers and 1387 heavy/light smokers are used in the present study). NAG-Aus/BigSib was used in the replication phase for ENGAGE^1^ (N = 1329 heavy/light smokers are used in the present study).

1. Thorgeirsson TE, Gudbjartsson DF, Surakka I, Vink JM, Amin N, et al. (2010) Sequence variants at CHRNB3-CHRNA6 and CYP2A6 affect smoking behavior. *Nat Genet* 42: 448-453.
2. The Tobacco and Genetics Consortium (2010) Genome-wide meta-analyses identify multiple loci associated with smoking behavior. *Nat Genet* 42: 441-447.
